# Supplementary material for: Heavy metal removal from coal fly ash for low carbon footprint cement
Source: Commun Eng. 2023 Mar 27;2:13. doi: 10.1038/s44172-023-00062-7 (PMC10955974; doi:10.1038/s44172-023-00062-7)
Supplement: Supplementary file 2 — Supplementary Information [file 44172_2023_62_MOESM2_ESM.pdf]

Supplementary Information

Heavy metal removal from coal fly ash for low carbon footprint cement

Bing Deng,[1,#,*] Wei Meng,[2,#] Paul A. Advincula,[1] Lucas Eddy,[1,3] Mine G. Ucak-Astarlioglu[4], Kevin M. Wyss,[1] Weiyin Chen,[1] Robert A. Carter,[1] Gang Li,[1] Yi Cheng,[1] Satish Nagarajaiah,[2,5,6,7,*] and James M. Tour[1,5,6,8,*]

[1]Department of Chemistry, Rice University, 6100 Main Street, Houston, Texas 77005, USA

[2]Department of Civil and Environmental Engineering, Rice University, 6100 Main Street, Houston, Texas 77005, USA

[3]Applied Physics Program, Rice University, 6100 Main Street, Houston, Texas 77005, USA

[4]Geotechnical and Structures Laboratory, U.S. Army Engineer Research and Development Center, Vicksburg, Mississippi, 39180-6199, USA

[5]Smalley-Curl Institute, Rice University, 6100 Main Street, Houston, Texas 77005, USA

[6]Department of Materials Science and NanoEngineering, Rice University, 6100 Main Street, Houston, Texas 77005, USA

[7]Department of Mechanical Engineering, Rice University, 6100 Main Street, Houston, Texas 77005, USA

[8]NanoCarbon Center and the Welch Institute for Advanced Materials, Rice University, 6100 Main Street, Houston, Texas 77005, USA

[*]Corresponding authors: J.M.T. (tour@rice.edu), S.N. (satish.nagarajaiah@rice.edu), B.D. (bingdeng@rice.edu)

[#] Those authors contributed equally.

Supplementary Note 1. Calculation of the removal efficiencies of heavy metals.

Considering that the mass of CFA used for FJH is *m*(CFA), the concentration of heavy metals in CFA is measured as *c*(CFA), the mass of CB used for FJH is *m*(CB), the concentration of heavy metals in CB is measured as *c*(CB), the mass of the remaining solid (mixture of CFA and residual carbon) after FJH is *m*(CFA+CB), and the concentration of heavy metals in the remaining solid is measured as *c*(CFA+CB), the removal efficiency (*R*) by FJH is calculated using the following equation,

$$R = \frac{c(\mathrm{CFA})\times m(\mathrm{CFA})+c(\mathrm{CB})\times m(\mathrm{CB}) - c(\mathrm{CFA+CB})\times m(\mathrm{CFA+CB})}{c(\mathrm{CFA})\times m(\mathrm{CFA})+c(\mathrm{CB})\times m(\mathrm{CB})}\times 100\% \quad (1)$$

Similarly, when metcoke (MC) is used as the conductive additives, the removal efficiency is calculated by,

$$R = \frac{c(\mathrm{CFA})\times m(\mathrm{CFA})+c(\mathrm{MC})\times m(\mathrm{MC}) - c(\mathrm{CFA+MC})\times m(\mathrm{CFA+MC})}{c(\mathrm{CFA})\times m(\mathrm{CFA})+c(\mathrm{MC})\times m(\mathrm{MC})}\times 100\% \quad (2)$$

When Plastic Ash (PA) is used as the conductive additives, the removal efficiency is calculated by,

$$R = \frac{c(\mathrm{CFA})\times m(\mathrm{CFA})+c(\mathrm{PA})\times m(\mathrm{PA}) - c(\mathrm{CFA+PA})\times m(\mathrm{CFA+PA})}{c(\mathrm{CFA})\times m(\mathrm{CFA})+c(\mathrm{PA})\times m(\mathrm{PA})}\times 100\% \quad (3)$$

And when BR is used as the raw material, the removal efficiency is calculated by,

$$R = \frac{c(\mathrm{BR})\times m(\mathrm{BR})+c(\mathrm{CB})\times m(\mathrm{CB}) - c(\mathrm{CFA+CB})\times m(\mathrm{CFA+CB})}{c(\mathrm{BR})\times m(\mathrm{BR})+c(\mathrm{CB})\times m(\mathrm{CB})}\times 100\% \quad (4)$$

Supplementary Note 2. Strategy for scaling up the FJH process.

The FJH process is highly scalable, as we demonstrated in our previous work[1]. Here, we first obtained the theoretical scaling rule of the process. Then, we demonstrated the scaleup of the sample to g size per batch. We further show that the productivity up to kg scale is realized in our

research lab. Last, we will present a brief discussion on the ongoing application of the FJH process at the industrial scale.

2.1 Scaling rule of FJH process by theoretical analysis.

For the evaporative separation process, the removal efficiencies of the heavy metals rely on the maximum temperature; hence, the available temperature across the sample is critical when scaling up the FJH process. For the Joule heating process, the heat amount (Q) is calculated by Supplementary Eq. 5,

$$Q = I^2 R t \quad (5)$$

where I is the current passing through the sample, R is the resistance, and t is the discharge time. The heat amount per volume (Q_v) is calculated by Supplementary Eq. 6,

$$Q_\mathrm{v} = j^2 \rho_\mathrm{e} t \quad (6)$$

where j is the current density, and ρ_e is the electrical resistivity.

The temperature change (ΔT) is proportional to the heat amount by Supplementary Eq. 7,

$$Q = C_\mathrm{p} m \Delta T \quad (7)$$

where C_p is heat capacity and m is the mass of the sample. The above equation can be reformulated per volume to Supplementary Eq. 8,

$$Q_\mathrm{v} = C_\mathrm{p} \rho_\mathrm{m} \Delta T \quad (8)$$

where ρ_m is the density of the sample. For a specific sample, the C_p and ρ were constant; hence, maintaining a constant Q_v is critical to keep the same temperature during FJH.

For a specific sample, the electrical resistivity (ρ_e) is constant; hence, to maintain a constant Q_v when increasing the sample mass, it is necessary to keep a constant j, according to Equation S6.

The charge amount (q) in the capacitor bank can be calculated by Supplementary Eq. 9,

$$q = CV \quad (9)$$

where C is the capacitance of the capacitor bank, and V is the voltage of the capacitor bank. Assuming that all the charges in the capacitor bank are discharged within the time t, the current (I) through the sample can be calculated by Supplementary Eq. 10,

$$I = \frac{q}{t} \quad (10)$$

As a result, the current density (j) can be calculated by Supplementary Eq. 11,

$$j = \frac{I}{S} = \frac{CV}{St} \quad (11)$$

where S is cross-sectional area of the sample. Since we usually use a cylinder-shaped sample, the mass (m) can be calculated by Supplementary Eq. 12,

$$m = \rho_{\mathrm{m}} SL \quad (12)$$

where ρ_{m} is the density of the sample, S is the cross-sectional area of the sample, and L is the length of the sample. For a specific sample type, the density (ρ_{m}) is kept the same.

To summarize, we can obtain Supplementary Eq. 13 that determines the current density,

$$j = \frac{CV\rho_{\mathrm{m}}L}{mt} \quad (13)$$

As we have shown above, when increasing the mass (m) of the sample, the current density (j) passing through the sample should be kept constant. This can be realized by the following measures: (1) increasing the FJH voltage (V), and/or (2) increasing the capacitance (C).

2.2 Scaling up to gram-scale per batch.

In our first-generation FJH setup, we used a capacitor bank composed of 10 aluminum electrolytic capacitors (450 V, 6 mF, Mouser #80-PEH200YX460BQU2) with a total capacitance of C_0 = 0.06 F (Supplementary Fig. 20a). In a small-scale experiment, the FJH voltage of V_0 = 120 V and capacitance of C_0 = 0.06 F were used for a sample mass of m_0 = 0.15 g. Here, we

demonstrate the scaling up of the FJH to a mass of m_1 = 3 g. We built a second-generation FJH setup with larger capacitance of C_1 = 0.624 F (Supplementary Fig. 20a). According to Supplementary Eq. 12, we obtain Supplementary Eq. 14,

$$\frac{m_1}{m_0} = \frac{C_1 V_1}{C_0 V_0} \quad (14)$$

For a mass of m_1 = 3 g and C_1 = 0.624 F, we used a FJH voltage of V_1 = 250 V, thus basically fitting with Supplementary Eq. 14. Further optimization of the FJH process, including the conductive additive content, the FJH voltage, and FJH time, is needed when scaling up the process. This is beyond our current research capabilities.

2.3 Scaling up to kg-scale in our research lab.

The FJH process was invented by our group for the conversion of carbon sources into flash graphene[2]. Since then, in our research laboratory, we built a continuous system for the FJH process, and achieved a production rate of >10 kg day^{-1} of graphene from metcoke. The flash temperature in that process was 3100 °C, similar to this work here. The FJH process for heavy metal removal from CFA is much the same as the graphene synthesis process; hence, the devices and processes developed could potentially be used for the purpose of CFA purification.

2.4 The conceptual design of the continuous FJH process.

The FJH process could be integrated with industrially available process for continuous running. For example, we here proposed a design combining FJH and belt roller. As shown in Supplementary Fig. 21, a sheet metal belt controlled by the tensioning roller is used for converting the CFA raw materials. The doctor blade is used to control the thickness and the compactness of the CFA raw materials. During FJH, since the sheet metal is not a good electrical conductor along

its length, the current will be concentrated where the electrodes are located. The FJH zone is placed in a vacuum chamber to collect the volatiles.

2.5 The FJH process is applied in industrial scale for the production of graphene.

The FJH process is undergoing industrial-scaled up by Universal Matter, Inc (https://www.universalmatter.com/) targeting the production rate of 100 ton day^{-1} in early 2024. The equipment and processes designed and optimized for the flash graphene synthesis are readily to be transferred for the heavy metal removal from CFA purpose.

Supplementary Note 3. The energy cost evaluation.

The electrical energy consumption is calculated by,

$$E = \frac{(V_1^2 - V_2^2) \times C}{2 \times M} \quad (15)$$

Where E is the energy per g (kJ g^{-1}), V_1 and V_2 are the voltage before and after FJH, respectively, C is the capacitance (C = 60 mF), and M is the mass per batch.

For a typical trial with V_1 = 120 V, V_2 = 70 V, and M = 0.15 g, the energy is calculated to be:

E = 1.90 kJ g^{-1} = 532 kWh ton^{-1}

Given that the industrial price of electric energy in Texas, USA is \$0.04 kWh^{-1}, the energy cost for treatment of CFA would be P(Energy) = \$21 ton^{-1}.

The materials cost is mostly from the conductive additives. Here, we used 33% metcoke as a conductive additive; 1 ton of the mixture contains 0.33 ton of metcoke. 92% of the metcoke could be recovered and reused, so the consumed metcoke is 0.026 ton. The bulk price of metcoke is ~\$150 ton^{-1} (ref[3]). Hence, the material cost for treatment of 1 ton of CFA would be P(Material) = \$4.0 ton^{-1}. Hence, when metcoke is used as the conductive additive, the total cost would be P(total)

= \$25 ton^{-1}. In contrast, when pyrolysis ash is used as the conductive additive and assuming a zero cost for pyrolysis ash, the total cost for treatment of CFA would be P = \$21 ton^{-1}.

Supplementary Note 4. Life cycle analysis.

4.1 Goal and scope.

This study is conducted under the requirements of ISO 14044 (ref[4]). The goal of this study is to compare the potential environmental impact of the current waste management of CFA (specifically, landfilling) with the reuse of the CFA as alternative cementitious materials after heavy metal removal by FJH. In particular, the analysis aims at verifying whether the GHG emissions and heavy metal emissions is reduced by the newly established FJH and partial substitution strategy, as well as its energy consumption demands.

4.2 Scenario description and system boundaries.

Four scenarios were considered in this study (Supplementary Fig. 22). In all the scenarios, 1 ton of cementitious materials for service life is used as the baseline and all other materials flow are normalized according to the cementitious materials (Supplementary Table 3).

Scenario 1 Landfilling: In this scenario, the pure OPC (1 ton) is used for the service life in cement, and the CFA (0.47 ton) and Plastic Ash (0.23 ton) are landfilled. Note that the mass of CFA and Plastic Ash are assigned according to their consumption in the FJH-Separation-Substitution scenario (see details in Scenario 3).

Scenario 2 Direct Substitution: In our cement application (Figs. 4b-c), the OPC is substituted with 30 wt% raw CFA. Hence, in this scenario, the OPC (0.7 ton) is mixed with raw CFA (0.3 ton) for the service life in cement. In this scenario, the Plastic Ash (0.23 ton) and part of

CFA (0.17 ton) is landfilled to compensate the materials consumption in Scenario 3. The landfill energy consumption is the same as Scenario 1.

Scenario 3 FJH-Separation-Substitution: In this scenario, the Plastic Ash is used as the conductive additive. The CFA (0.47 ton) is mixed with 33 wt% Plastic Ash (0.23 ton), and the 0.7 tons undergo FJH to remove the heavy metals, which leading to a mixture of purified CFA-Plastic Ash (0.33 ton). The residual carbon in the mixture of purified CFA-Plastic Ash is separated by sieving (Supplementary Fig. 9), yielding the purified CFA (0.3 ton). In our cement application (Figs. 4b-c), the OPC is substituted with 30 wt% purified CFA. Hence, the OPC (0.7 ton) is mixed with the purified CFA (0.3 ton) as the service life in cement. No material is landfilled in this scenario.

Scenario 4 FJH-Substitution: In this scenario, the Plastic Ash is used as the conductive additive. The CFA (0.07 ton) is mixed with 33 wt% Plastic Ash (0.04 ton), and the 0.11 ton undergo FJH to remove the heavy metals, which leading to a mixture of purified CFA-Plastic Ash (0.05 ton). In our cement application (Supplementary Fig. 18), the OPC is substituted with 5 wt% purified CFA-Plastic Ash without the separation process. Hence, the OPC (0.95 ton) is mixed with the purified CFA-Plastic Ash (0.05 ton) for the service life in cement. In this scenario, some portion of CFA (0.4 ton) and Plastic Ash (0.19 ton) are landfilled to compensate the materials consumption in Scenario 3.

4.3 Life cycle inventory.

The environmental impacts, including GHG emission and heavy metal emission, and energy consumption demand, for the raw materials production, processing, and landfilling are summarized in Supplementary Table 4. The values are explained below.

Materials production: The GHG emission (849.50 kg ton^{-1}) and energy consumption (4581 MJ ton^{-1}) for OPC is from the Argonne GREET model[5]. The GHG emission (10.1 kg ton^{-1}) and energy consumption (199 MJ ton^{-1}) of CFA is from a literature[6]. The GHG emission and energy consumption of Plastic Ash is unavailable; here, the biomass ash data are used as an alternative due to their similar process (GHG emission at 0.028 kg ton^{-1}, energy consumption at 5.94 MJ ton^{-1})[6].

Service life as cement: We assume that the heavy metal emissions to the surrounding environments take place in the service life of cement. The heavy metal emission is defined as the total leaching contents of As, Co, and Ni. The leachable As, Co, and Ni contents in cement reach a plateau according to the acid leaching experiments (Figs. 4d-f); hence, the leachable heavy metal content at 125 h is used as the heavy metal emission in the service life of cement. Hence, the heavy metal emissions for pure OPC, raw CFA, and purified CFA are designated to be 2.25 g ton^{-1}, 1.88 g ton^{-1}, and 0.94 g ton^{-1}, respectively. When a mixture of cement composite is used, the heavy metal emission is normalized according to their mass ratio. Therefore, the heavy metal emission for the OPC substituted with 30 wt% raw CFA, the OPC substituted with 30 wt% purified CFA, and the OPC substituted with 5% purified CFA are calculated to be 2.14 g ton^{-1}, 1.85 g ton^{-1}, and 2.18 g ton^{-1}, respectively.

Landfilling: We assume that heavy metal emissions take place in the landfilling of CFA. The heavy metal emission from CFA landfilling is defined as the total leachable contents of As, Co, and Ni, which are assigned as the plateau content at 125 h, i.e., 1.88 g ton^{-1}, in the acid leaching experiments (Figs. 4d-f). We do not consider the heavy metal emission from Plastic Ash landfilling in this work. Since CFA is mostly composed of inorganics and Plastic Ash is mostly composed of graphitized carbon that is very stable in environment, we here assume no GHG emission during

the landfilling of CFA and Plastic Ash. Energy input is necessary for the landfilling of CFA and Plastic Ash. According to a recent literature[7], the landfill energy consumption is ~19.6 MJ ton^{-1}, including human labor, diesel fuel, transportation, electricity, *etc*. This energy is assigned to the energy consumption of CFA and Plastic Ash landfilling.

Processing - Mixing: Energy input is needed for the mixing process, including the mixing of raw CFA and Plastic Ash for FJH, and the mixing of non-purified or purified CFA and OPC for cement. We assume that the mixing is conducted using a electricity driven Powder Mixer[8] with the energy consumption of 9.432 MJ ton^{-1}. No GHG emission and heavy metal emission in the mixing process.

Processing - FJH: The energy consumption for FJH is estimated to be 2901.6 MJ ton^{-1}, according to the detailed discussion in Supplementary Note 3. Heavy metal is collected in the FJH process thus no heavy metal emission at this step.

Processing – Separation by sieving: We assume that the separation is conducted using an industrial vibrating sieving machine. For a typical shaker machine, the capacity is estimated to be m = 0.034 ton according to its volume. The powder is 80 W. If we assume the processing time of 0.5 h, the separation energy consumption is estimated to be ~4 MJ ton^{-1}. The separation process has no heavy metal or GHG emissions.

4.4 Life cycle impact assessment.

In this study, the environmental impacts were categorized into 3 midpoint indicators, including heavy metal emissions (Supplementary Table 5), GHG emissions (Supplementary Table 6), and energy consumptions (Supplementary Table 7).

4.5 Sensitivity and uncertainty.

First, due to the data availability, the energy consumption and GHG emission values for materials involved in this study are from different sources, which could introduce some uncertainty. Second, several assumptions were made in this study about the release of heavy metals into environments, the materials mixing process, and the separation process. Third, even though we proposed the scalability of the FJH process (Supplementary Note 2), the FJH process realized in this work is on the gram scale; hence, there will be uncertainty in energy consumption when scaling up the FJH to ton scale.

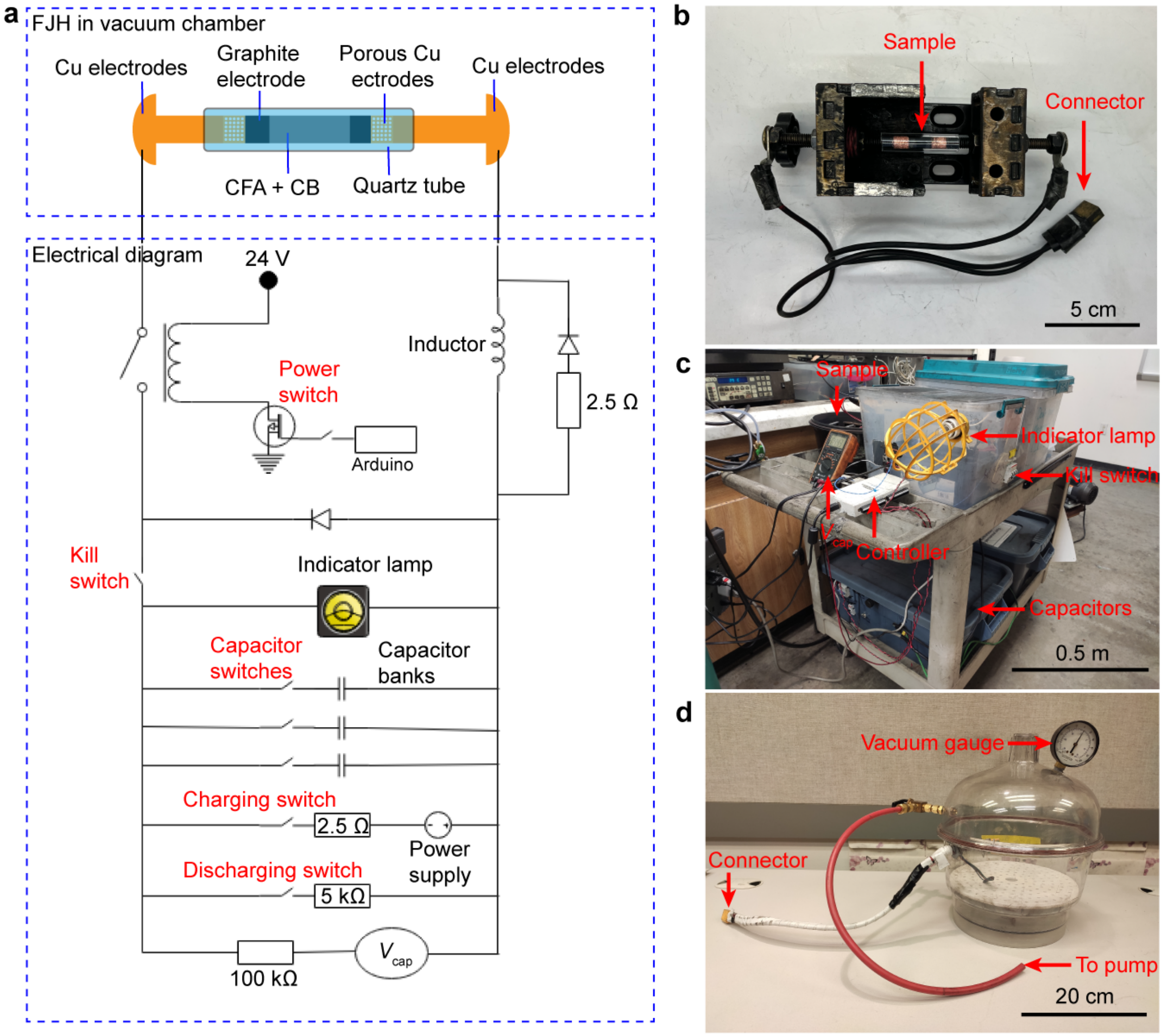

Supplementary Fig. 1. The electrical diagram and pictures of the flash Joule heating (FJH) system. (**a**) Electrical diagram of the FJH system. The capacitor bank is composed of 10 aluminum capacitors (450 V, 6 mF, Mouser #80-PEH200YX460BQU2). The total capacitance is 60 mF. The detailed description of the electrical components could be found in our previous publications[1,9-11]. The graphite electrodes are loosely fitted in the quartz tube, and the porous Cu electrodes made from compressed Cu wool are used to permit the outgassing. CFA, coal fly ash; CB, carbon black. (**b**) FJH reaction stage. (**c**) Picture of the FJH setup. (**d**) FJH mild vacuum chamber made from a plastic vacuum desiccator.

***CAUTION**: There is a risk of electrical shock if improperly operated. We recommend the following safety guidance when using this equipment. More safety practices can be found in our previous publications*[1,9-11].

1. Enclose or carefully insulate all wire connections.
2. All connections, wires, and components must be suitable for the high voltages and currents.
3. One hand rule. Use only one hand when working on the system, with the other hand not touching any grounded surface.
4. Provide a mechanical discharge circuit breaker switch connected to a power resistor of a few hundred ohms to rapidly bleed off the capacitor charge.
5. Provide a "kill" circuit breaker switches to disconnect the sample holder from the capacitor bank.
6. Post high voltage warning signs on the apparatus.
7. Keep in mind that the system can discharge many thousands of Joules in milliseconds, which can cause components such as relays to explore.
8. Keep a voltmeter with high voltage test leads nearby at all times. When working on the capacitor bank, always check the voltage on each.
9. Wear thick rubber gloves when using the apparatus to protect from electrocution.
10. Welding safety glasses are recommended to block the infrared and ultraviolet light during the flashing reaction.
11. The reliability and robustness of the FJH system should be confirmed by an experienced electrical technician.
12. All users should be properly trained by an experienced electrical technician.
13. Reinspection by an electrical technician should occur weekly.

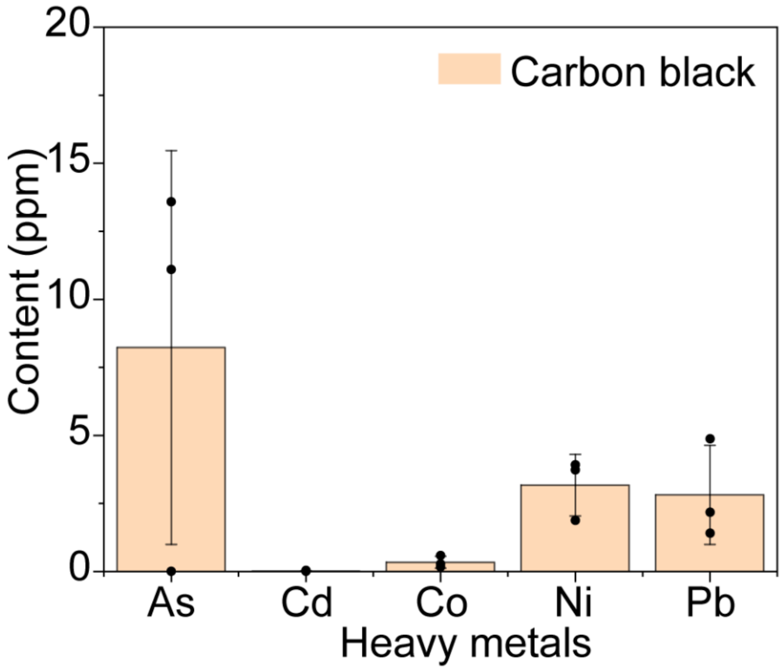

Supplementary Fig. 2. Heavy metal contents in carbon black. The error bars denote the standard deviation where n = 3.

Supplementary Discussion 1: The concentrations of heavy metal in CB are: As, ~8.23 ppm; Cd, 0.01 ppm; Co, ~0.34 ppm; Ni, ~1.13 ppm; and Pb, ~2.82 ppm. For comparison, the concentrations of heavy metal in CFA-F are: As, ~88.61 ppm; Cd, 0.62 ppm; Co, ~18.72 ppm; Ni, ~43.46 ppm; and Pb, ~28.33 ppm. The concentrations of heavy metal in CFA-C are: As, ~59.66 ppm; Cd, 0.76 ppm; Co, ~15.93 ppm; Ni, ~36.57 ppm; and Pb, ~22.84 ppm. Hence, the concentrations of heavy metal in CB are 1.7% to 9.9% of those in CFA-F, and 1.4% to 13.8% of those in CFA-C. As a result, the concentration of heavy metals in CB is statistically significant. Hence, in the calculation of the removal efficiencies of heavy metals, the total content of heavy metals in the combined CFA and CB is used as the baseline.

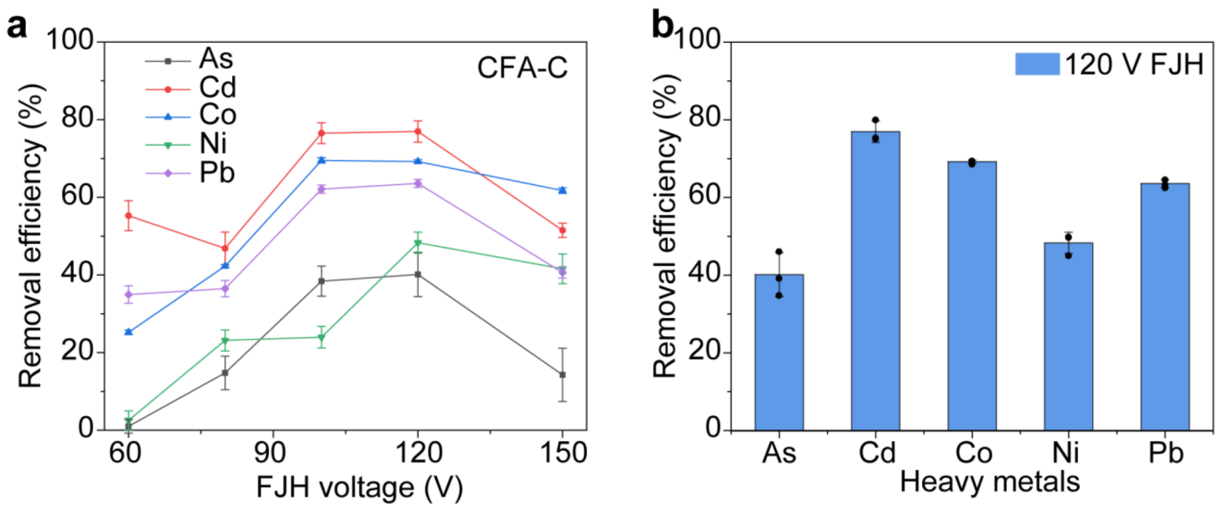

Supplementary Fig. 3. Removal of heavy metals from class C coal fly ash (CFA-C). (**a**) Removal efficiencies of heavy metals from CFA-C varied with the flash Joule heating (FJH) voltage. (**b**) Removal efficiencies of heavy metals from CFA-C at a FJH voltage of 120 V. The error bars in **a** and **b** denote the standard deviation where n = 3.

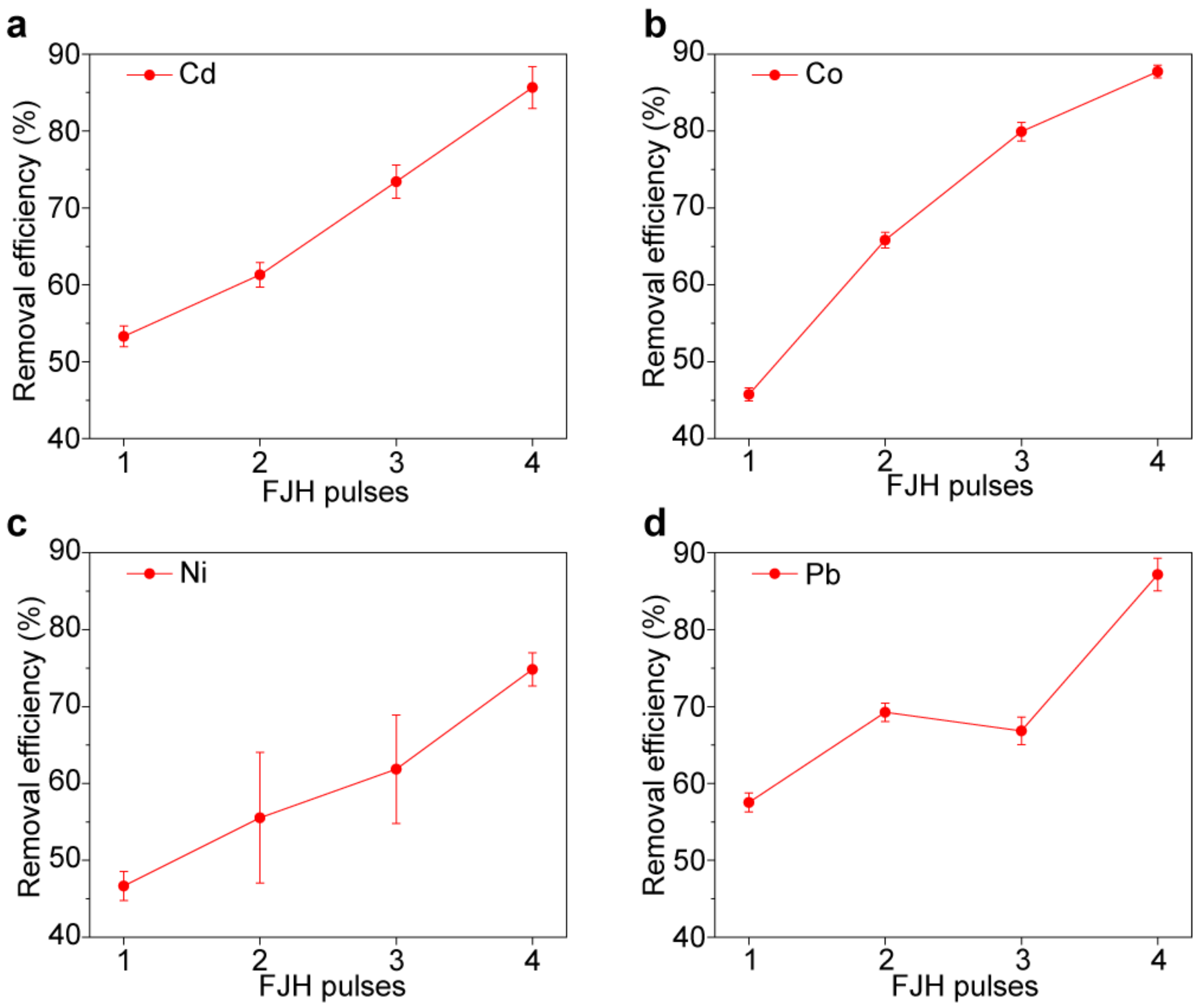

Supplementary Fig. 4. Improving the heavy metals removal efficiency of CFA-C by multiple flash Joule heating (FJH) pulses. (**a**) Cd removal efficiencies varied with FJH pulses. (**b**) Co removal efficiencies varied with FJH pulses. (**c**) Ni removal efficiencies varied with FJH pulses. (**d**) Pb removal efficiencies varied with FJH pulses. The error bars denote the standard deviation where n = 3.

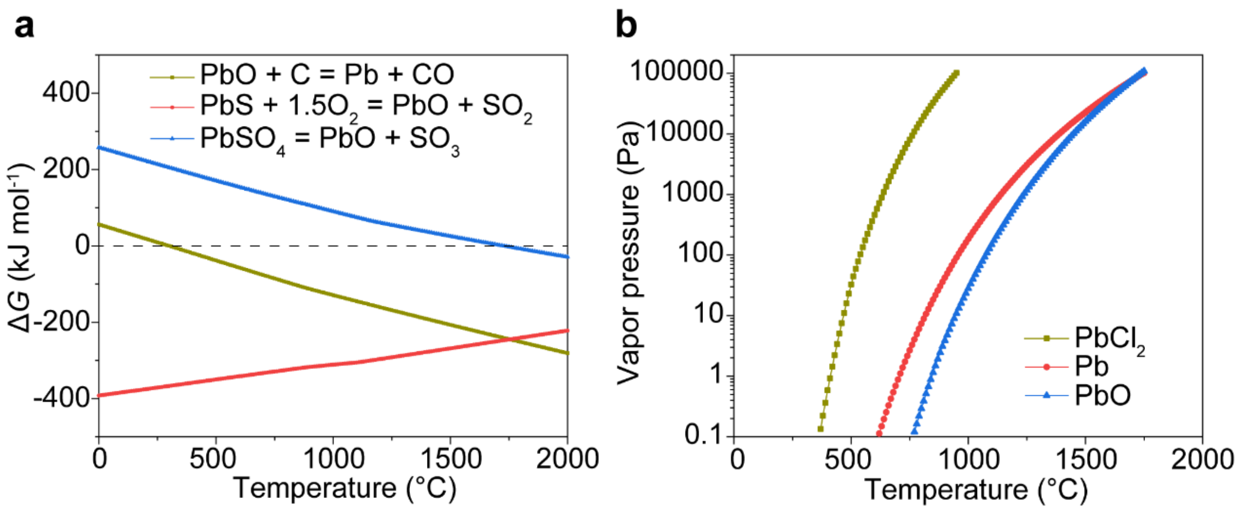

Supplementary Fig. 5. Effect of chemical state to the FJH process. (**a**) Thermodynamic analysis of carbothermic reduction of PbO and thermal decomposition of $PbSO_4$. The dash line indicates ΔG = 0 kJ mol^{-1}. (**b**) Vapor pressure-temperature relationships of Pb species.

Supplementary Discussion 2: The heavy metals in CFA are in natural ore or oxides forms. Depending on the reactivity and thermal stability of these species, there are several scenarios: (1) The heavy metal species directly evaporate; (2) The heavy metals species thermally decompose to other compounds and then evaporate; (3) The heavy metals compounds are carbothermic reduced to elemental metals and then evaporate. The FJH could achieve an ultrahigh temperature up to 3000 °C, which is higher than the temperature required for all above scenarios. We used the different speciation of lead (Pb, $PbCl_2$, PbO, PbS, and $PbSO_4$) as an example for the thermodynamic analysis. Pb and $PbCl_2$ could directly evaporate. PbO could directly evaporate, or it can be carbothermic reduced to Pb(0) by the Supplementary Equation 16:

PbO (s) + C (s) = Pb (s) + CO (g) (16)

PbS could be converted to PbO by the Supplementary Equation 17:

PbS (s) + 1.5O_2 (g) = PbO (s) + SO_2 (g) (17)

$PbSO_4$ could decompose to PbO by the Supplementary Equation 18:

$PbSO_4$ (s) = PbO (s) + SO_3 (g) (18)

We calculated the Gibbs free energy change of these reactions (Supplementary Fig. 5a) using the software HSC Chemistry 10. All these reactions are thermodynamically favorable below 2000 °C. Moreover, we calculated the vapor pressure varied with temperature for different Pb species (Supplementary Fig. 5b). All these Pb species have a high vapor pressure below 2000 °C. Since our FJH process can achieve a very high temperature of 3000 °C, all above chemical conversion and evaporation processes are thermodynamically favorable and probably rapid. Above all, we conclude that the FJH process is applicable to heavy metals removal regardless of their chemical states.

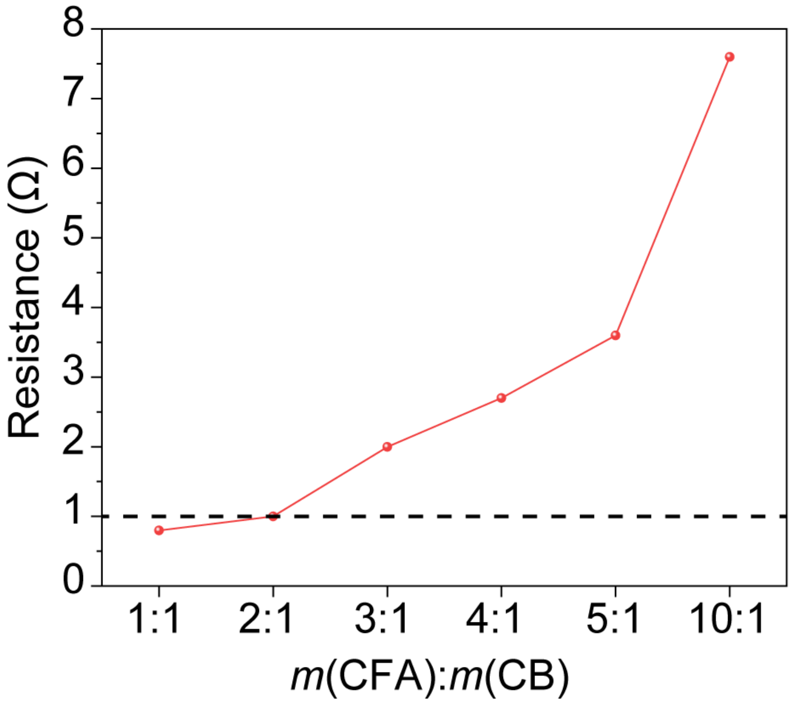

Supplementary Fig. 6. Sample resistance measurement. Sample resistance varied with mass ratio of coal fly ash (CFA) and carbon black (CB). The dash line indicates R = 1 Ω.

Supplementary Discussion 3: In our FJH process, the sample resistance is important: if the resistance is too high, the current is not large enough to generate heating; in contrast, if the resistance is too low, the sample resembles a conductor and cannot generate enough heat neither. According to our experience, a resistance of ~1 Ω is the best resistance for the FJH process.

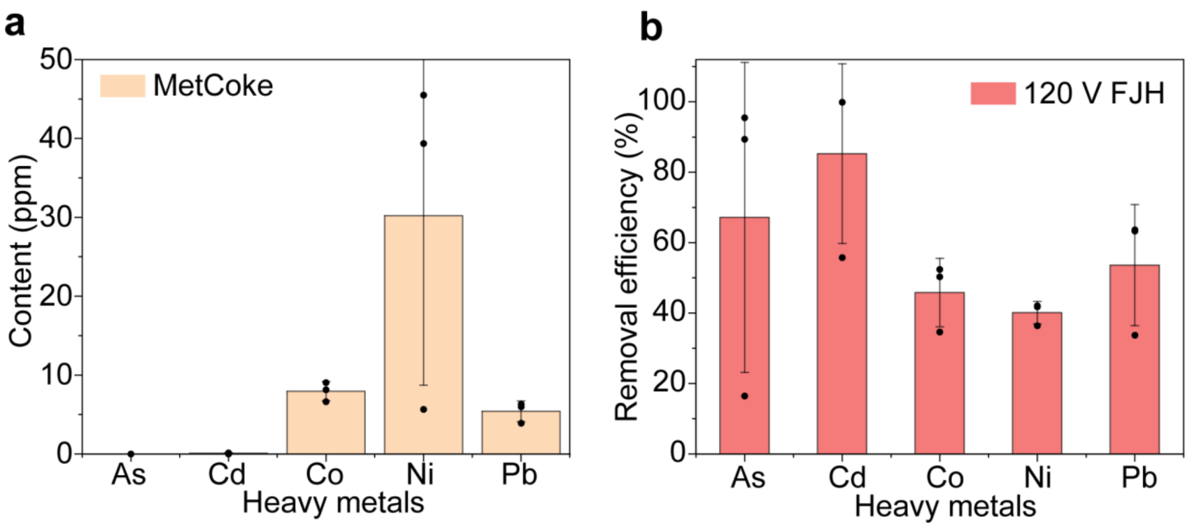

Supplementary Fig. 7. Removal of heavy metals from CFA-F using metallurgical coke (metcoke) as the conductive additive. (**a**) Heavy metal content in metcoke. (**b**) Removal efficiencies of heavy metals from CFA-F with a single flash Joule heating (FJH) voltage of 120 V and 1 s. The error bars denote the standard deviation where n = 3.

Supplementary Discussion 4: The concentrations of heavy metal in metcoke are: As, below the ICP MS detection limit; Cd, 0.09 ppm; Co of ~7.95 ppm; Ni of ~30.2 ppm; and Pb of ~5.4 ppm. For comparison, the concentrations of heavy metal in CFA-F are: As, ~88.61 ppm; Cd, 0.62 ppm; Co, ~18.72 ppm; Ni, ~43.46 ppm; and Pb, ~28.33 ppm. Hence, the concentrations of heavy metal in CB are 0–69 % of those in CFA-F. As a result, the concentration of heavy metals in CB is statistically significant. In the calculation of the removal efficiencies of heavy metals, the total content of heavy metal in the combined CFA and CB is used as the baseline. The removal efficiencies of heavy metal are somewhat smaller than those by using CB as additive (Figure 2f). CB has a better conductivity than that of metcoke (R ~1.0 Ω for CB, and R ~1.5 Ω for metcoke), thus the temperature would be higher for CB as conductive additive. In addition, CB has a much

smaller average particle size of ~10 nm, while metcoke has an average particle size <150 μm. The difference in particle size is the reason that the heating uniformity is better when using CB as conductive additives.

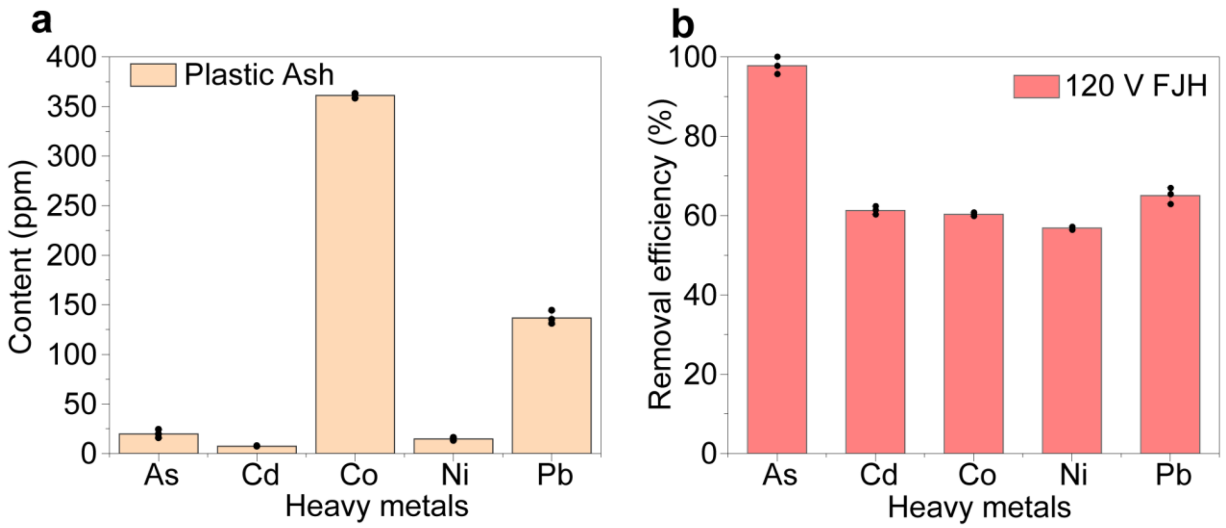

Supplementary Fig. 8. Removal of heavy metals from CFA-F using Plastic Ash as the conductive additive. (**a**) Heavy metal content in Plastic Ash. (**b**) Heavy metal removal efficiencies of from CFA-F with flash Joule heating (FJH) voltage of 120 V and 1 s. The error bars denote the standard deviation where n = 3.

Supplementary Discussion 5: The removal efficiencies of heavy metals from plastic pyrolysis ash are somewhat smaller than those by using CB as additive (Figure 2f). CB has a better conductivity than the Plastic Ash, with R ~1.0 Ω for CB as additive, and R ~3.0 Ω for Plastic Ash as additive, thus the temperature would be higher for CB as conductive additive. In addition, CB has a much smaller particle size and much higher surface area than Plastic Ash, and hence the heating uniformity would be better for CB as conductive additives.

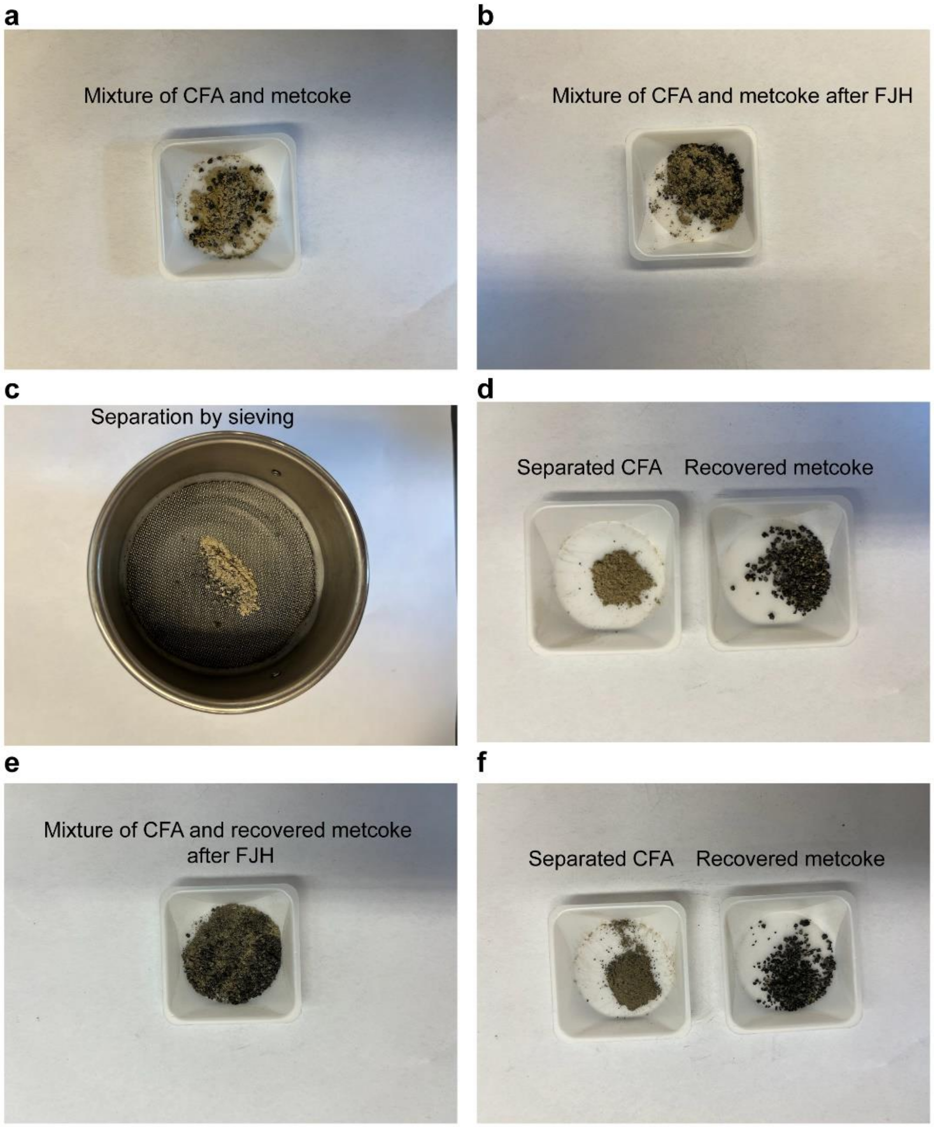

Supplementary Fig. 9. Separation and reuse of the carbon additives. (**a**) Picture of the mixture of coal fly ash (CFA) and metallurgical coke (metcoke). (**b**) Picture of the mixture of CFA and metcoke after flash Joule heating (FJH). (**c**) The separation of CFA and metcoke by sieving. (**d**) Pictures of the separated CFA and recovered metcoke. (**e**) Picture of the mixture of CFA and recovered metcoke after FJH. (**f**) Pictures of the separated CFA and recovered metcoke.

Supplementary Discussion 6: By using metcoke as an example, we demonstrated the separation of purified CFA and metcoke by sieving. The CFA has fine particle size, and we chose the metcoke with relatively large size. The mixture of CFA (~333 mg) and metcoke (~167 mg) were used (Supplementary Fig. 9a). After FJH, the particle size of CFA and metcoke remained almost unchanged (Supplementary Fig. 9b). Hence, we were able to separate CFA and metcoke by sieving (Supplementary Fig. 9c). In a typical process, the recovered mass of metcoke is *m*(recovered metcoke) = 154 mg; hence, we got the metcoke recovery yield of ~92% (Supplementary Fig. 9d).

The recovered metcoke could be reused for further FJH treatment. We used the recovered metcoke (154 mg) with some new metcoke (13 mg) as the conductive additives to purify the CFA (333 mg), as shown in Supplementary Fig. 9e. After the FJH process and subsequent separation by sieving, we recovered the metcoke with mass of *m*(recovered metcoke) = 156 mg and the metcoke recovery yield of ~93% (Supplementary Fig. 9f).

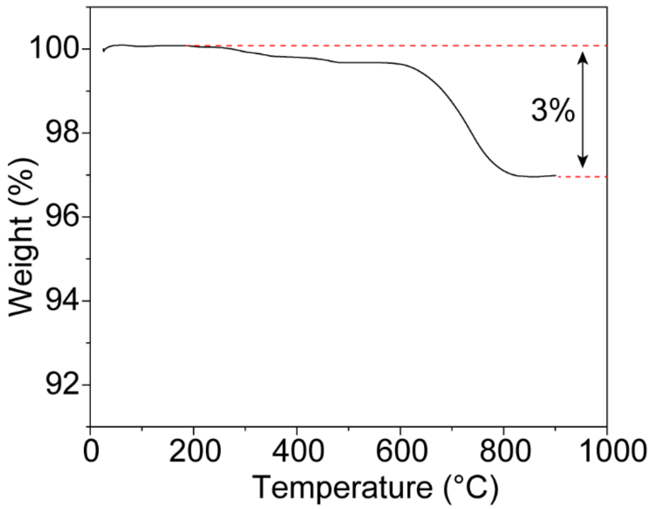

Supplementary Fig. 10. Residual carbon in the CFA sample after sieving separation. TGA curve of purified CFA after CB removal by sieving. TGA was conducted in air with the heating rate of 10 °C/min.

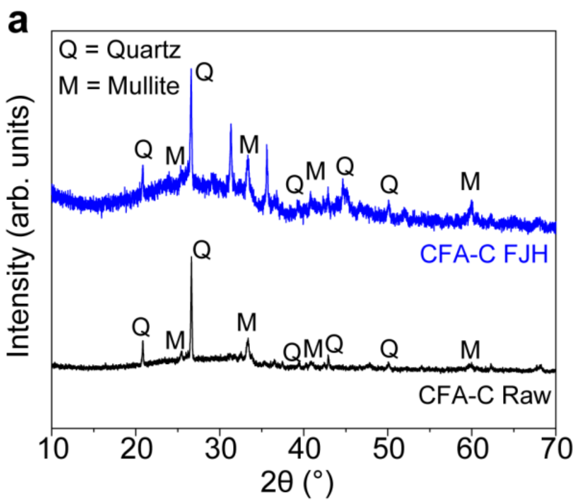

Supplementary Fig. 11. Characterization of coal fly ash (CFA) after flash Joule heating (FJH). (**a**) XRD patterns of class F coal fly ash (CFA-F) raw material and CFA-F after FJH. (**b**) XRD patterns of class C coal fly ash (CFA-C) raw material and CFA-C after FJH. Mullite (PDF 15-0776) and quartz (PDF 33-1161) are used as references.

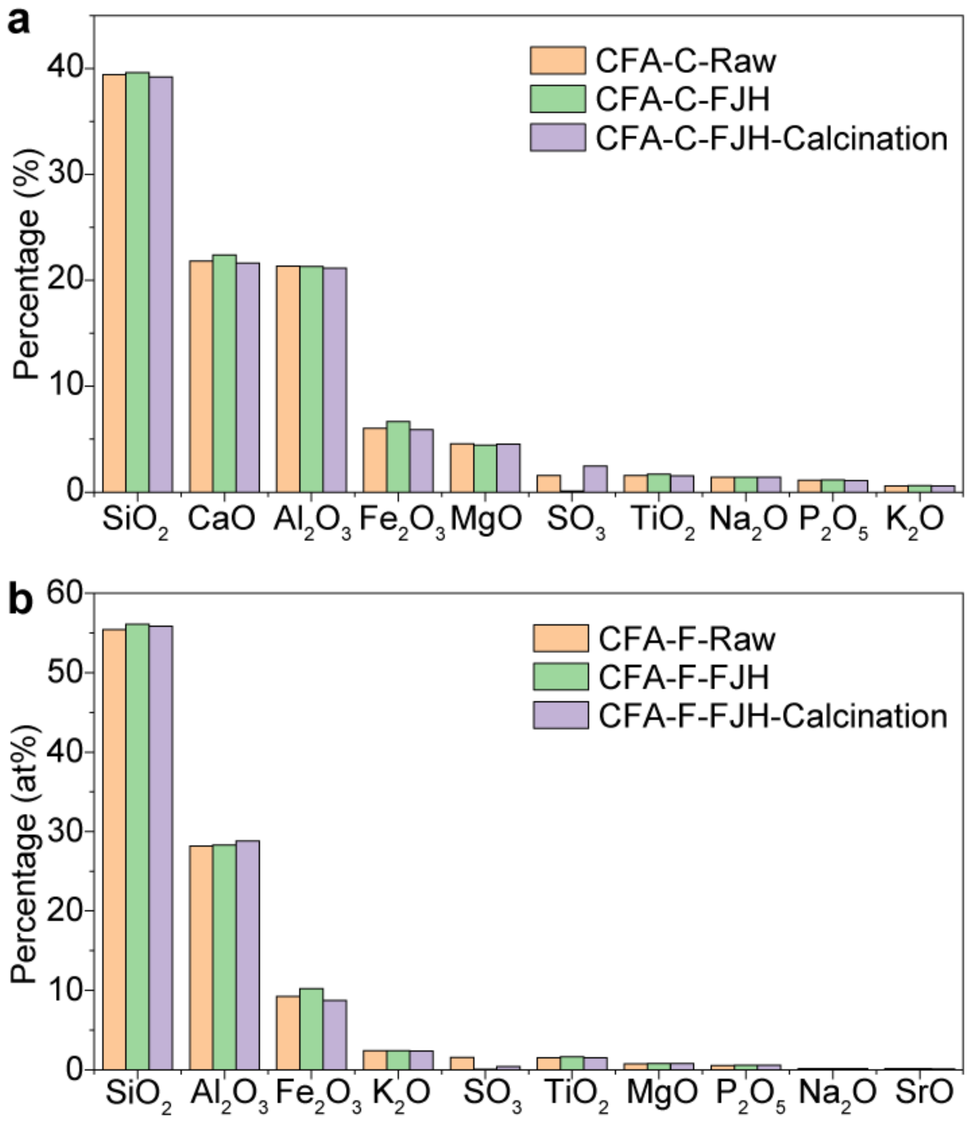

Supplementary Fig. 12. XRF characterization of coal fly ash (CFA). (**a**) Main inorganic component molar percentage of class C CFA raw material (CFA-C-Raw), CFA-C after flash Joule heating (CFA-C-FJH), and CFA-C after FJH and calcination (CFA-C-FJH-Calcination). (**b**) Main inorganic component molar percentage of class F CFA raw materials (CFA-F-Raw), CFA-F after FJH (CFA-F-FJH), and CFA-F after FJH and calcination (CFA-F-FJH-Calcination). The calcination was conducted in air at 700 °C for 1 h to remove the residual carbon.

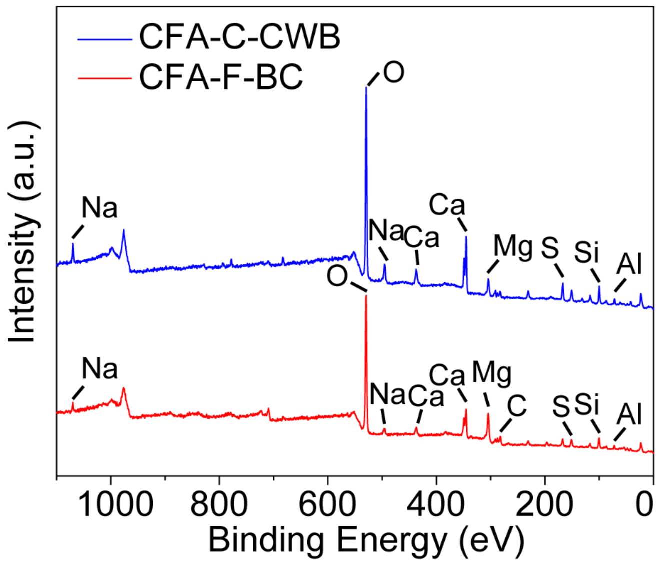

Supplementary Fig. 13. Element characterization of coal fly ash (CFA). XPS full spectra of class F CFA from Boral Cumberland (CFA-F-BC), and class C CFA from Charah White Bluff (CFA-C-CWB).

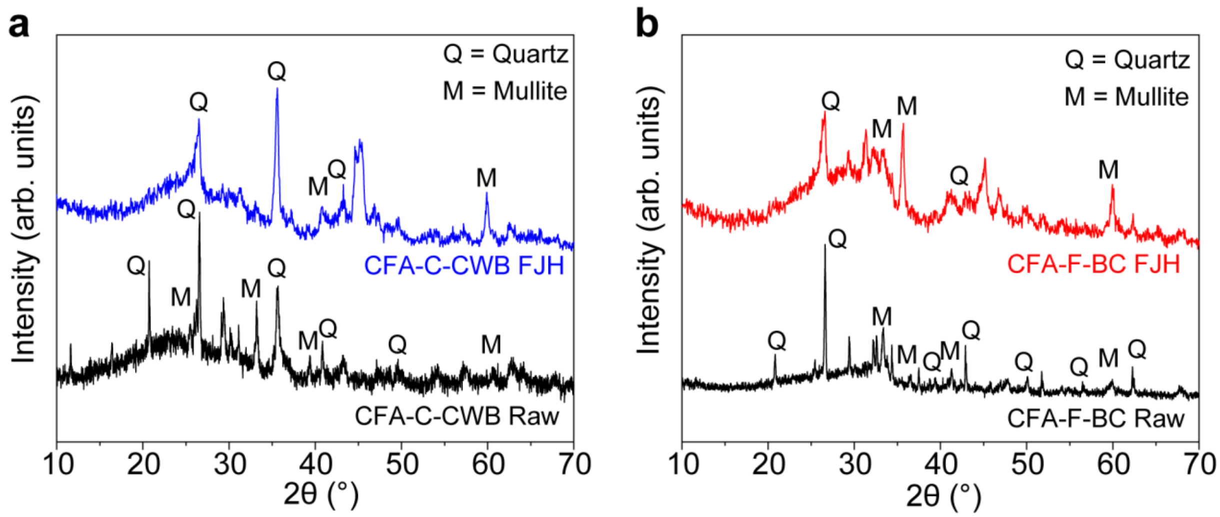

Supplementary Fig. 14. Characterization of coal fly ash (CFA) after flash Joule heating (FJH). (**a**) XRD patterns of class C CFA from Charah White Bluff (CFA-C-CWB) raw materials and CFA-C-CWB after FJH. (**b**) XRD patterns of class F CFA from Boral Cumberland (CFA-F-

BC) raw material and CFA-F-BC after FJH. Mullite (PDF 15-0776) and quartz (PDF 33-1161) are used as references.

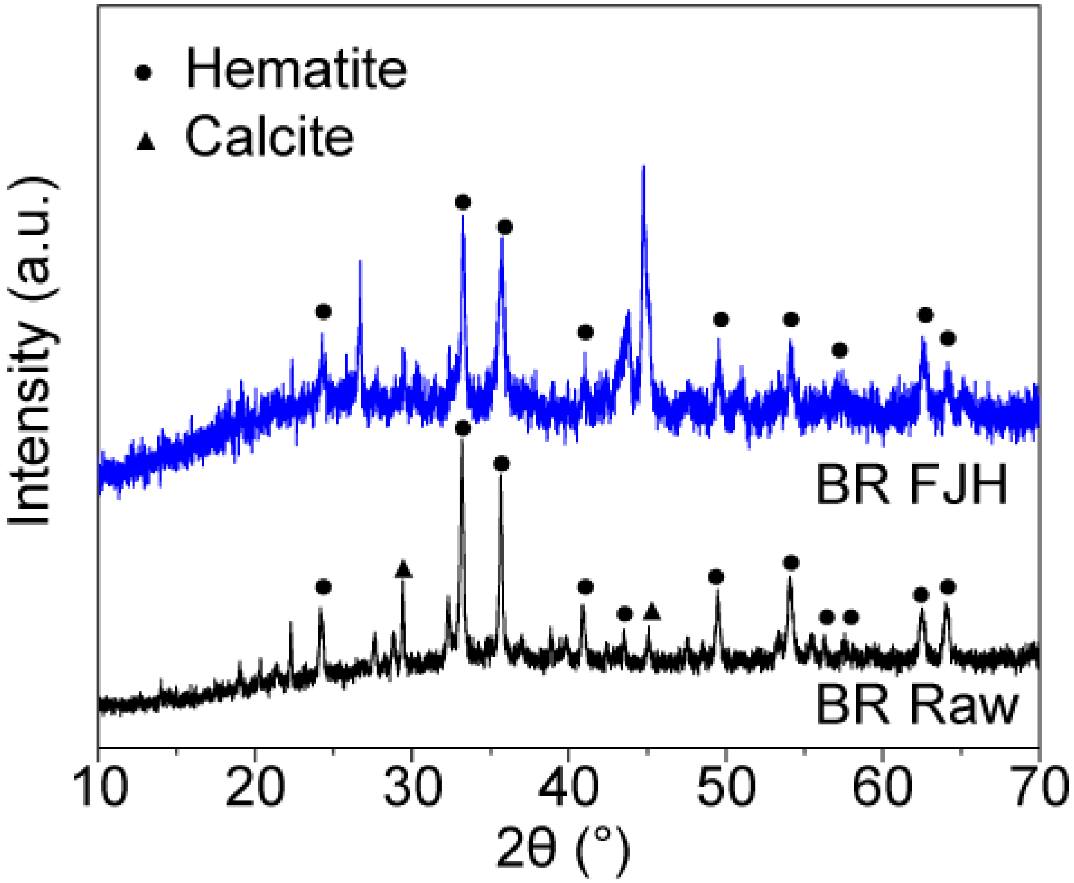

Supplementary Fig. 15. Characterization of BR after FJH. XRD patterns of bauxite residue (BR) raw materials and BR after flash Joule heating (FJH). Hematite (PDF 02-0919) and calcite (PDF 47-1743) are used as references.

Supplementary Discussion 7: In BR, hematite and calcite are the major crystal components. After FJH, the hematite remains and the calcite was reduced, presumably by the Supplementary Equation 19:

$CaCO_3 = CaO + CO_2$ (19)

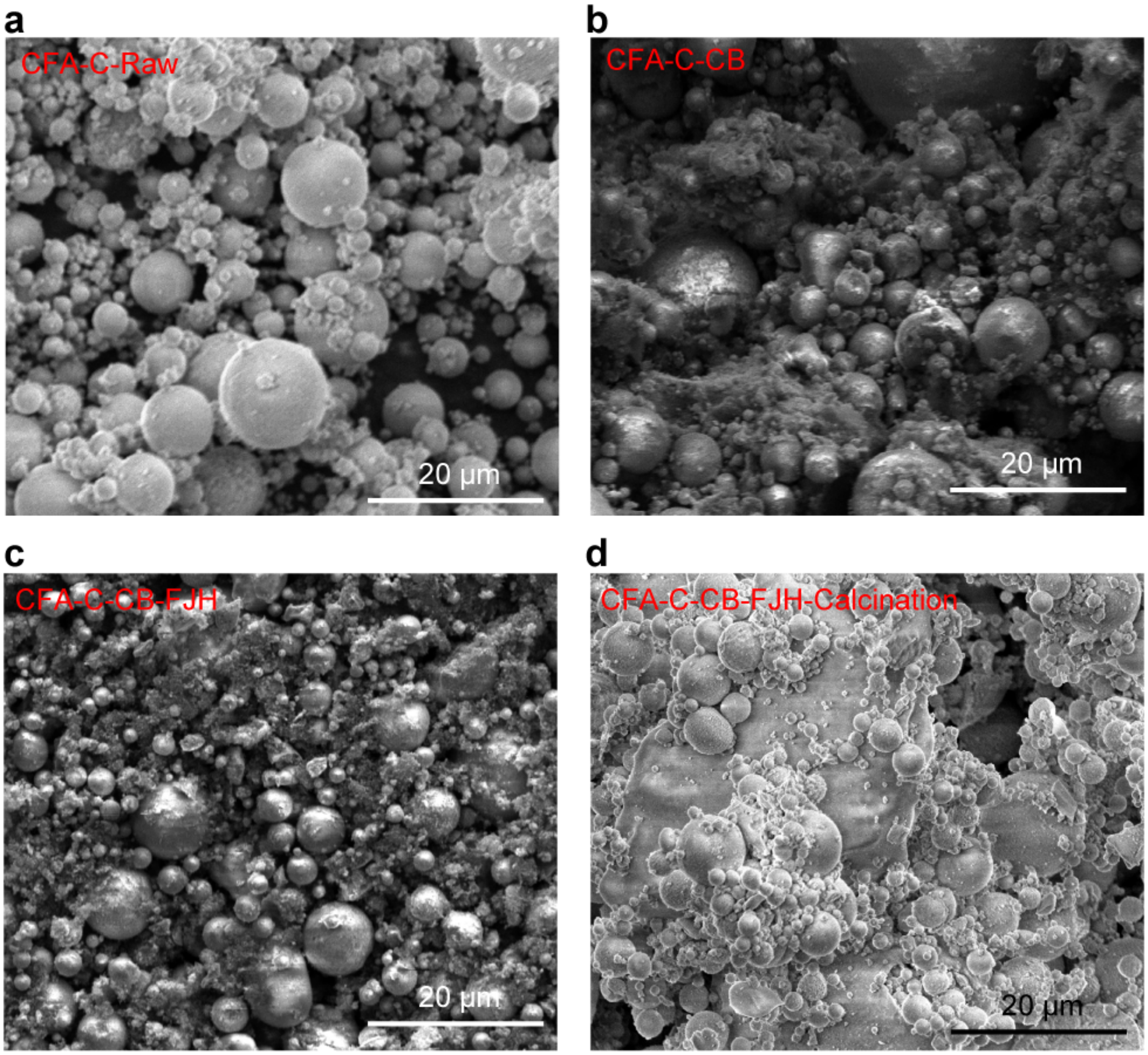

Supplementary Fig. 16. SEM characterization of class C coal fly ash (CFA-C). (**a**) SEM image of CFA-C Raw materials. (**b**) SEM image of the mixture of CFA-C and CB (CFA-C-CB). (**c**) SEM image of the CFA-C-CB after FJH (CFA-C-CB-FJH). (**d**) SEM image of the CFA-C-CB after FJH and calcination (CFA-C-CB-FJH-Calcination). The calcination was conducted in air at 700 °C for 1 h to remove the carbon residue.

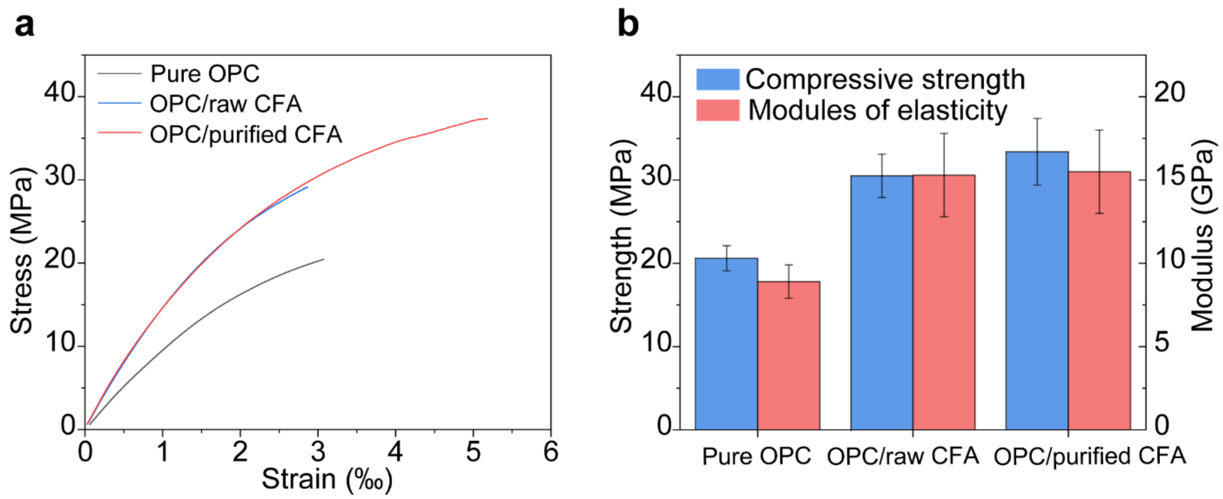

Supplementary Fig. 17. Measurement of purified coal fly ash (CFA) in cement composites after 1-day curing. (**a**) Representative stress-strain curves of the pure ordinary Portland cement (Pure OPC), the OPC substituted with 30 wt% raw CFA (OPC/raw CFA), and the OPC substituted with 30 wt% purified CFA (OPC/purified CFA). (**b**) Compressive strengths and moduli of elasticity statistics of the Pure OPC, the OPC/raw CFA, and the OPC/purified CFA. The error bars denote the standard deviation where n = 3.

Supplementary Discussion 8: The compressive strength of the OPC/purified CFA is 33.4 ± 4.0 MPa, exhibiting ~62% increase compared with that of the Pure OPC; the modulus of elasticity of OPC/purified CFA is 15.5 ± 2.5 GPa, which is ~74% higher than that of the pure OPC. In addition, the performance of OPC/purified CFA is comparable to those of the OPC/raw CFA.

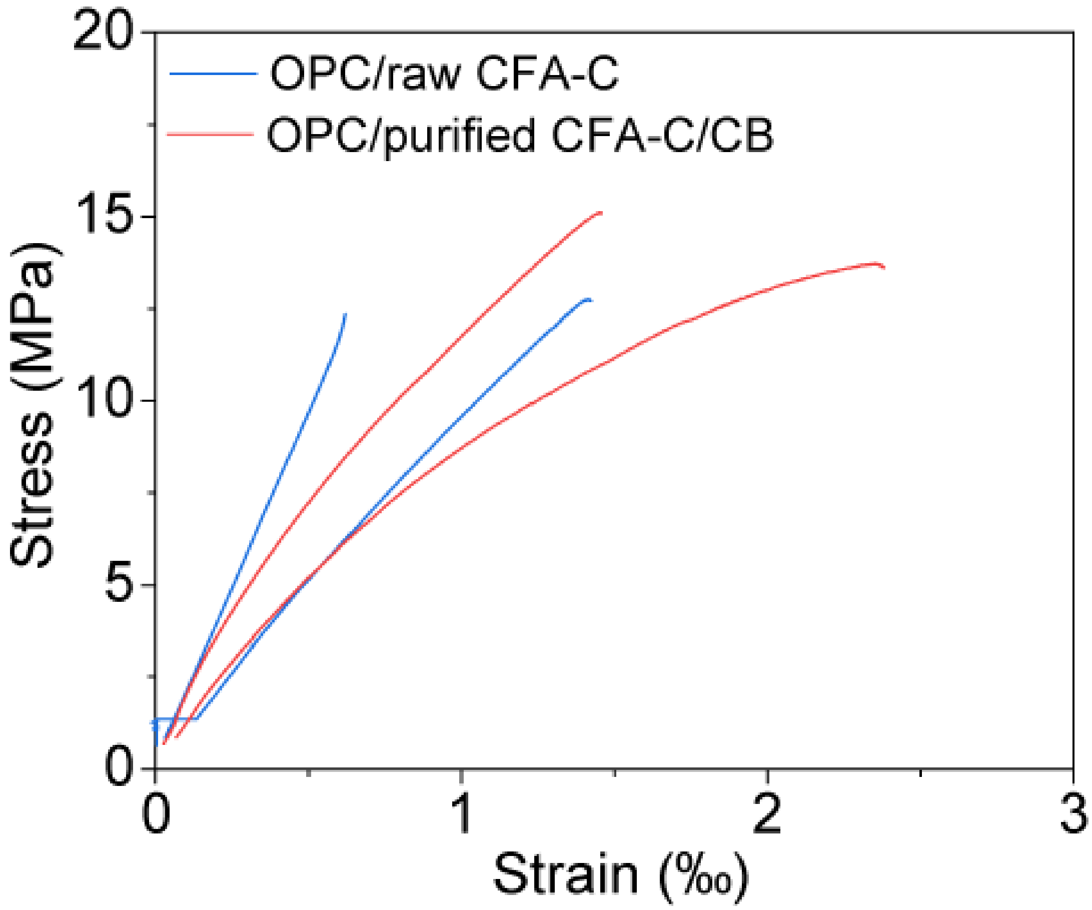

Supplementary Fig. 18. Application of purified class C coal fly ash (CFA-C) with carbon residue in cement. Stress-strain curves of the ordinary Portland cement (OPC) substituted with 5 wt% CFA-C raw materials (OPC/raw CFA-C), and the OPC substituted with 5 wt% purified CFA-C with residual carbon black (OPC/purified CFA-C/CB). Two independent experiments were conducted for the two different kinds of samples.

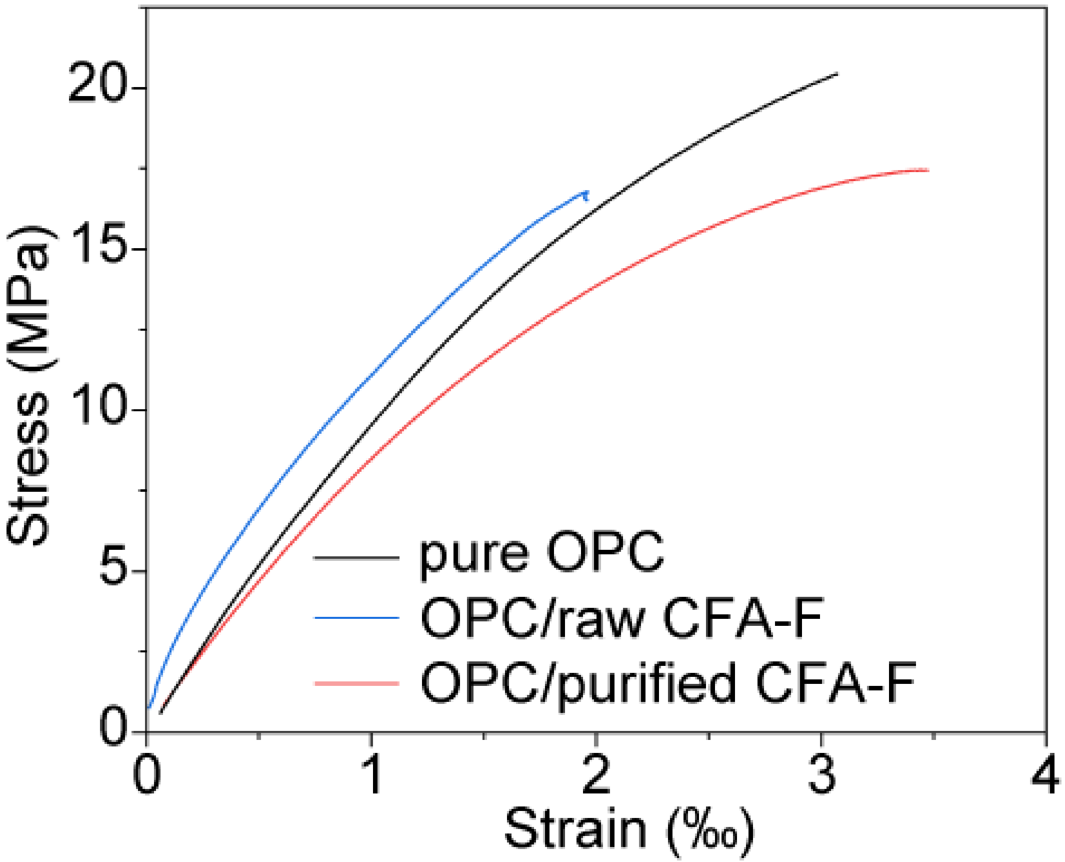

Supplementary Fig. 19. Application of purified class F coal fly ash (CFA-F) in cement. Stress-strain curves of the pure ordinary Portland cement (pure OPC), the OPC substituted with 30 wt% raw CFA-F (OPC/raw CFA-F), and the OPC substituted with 30 wt% purified CFA-F after removing the residual carbon by calcination (OPC/purified CFA-F).

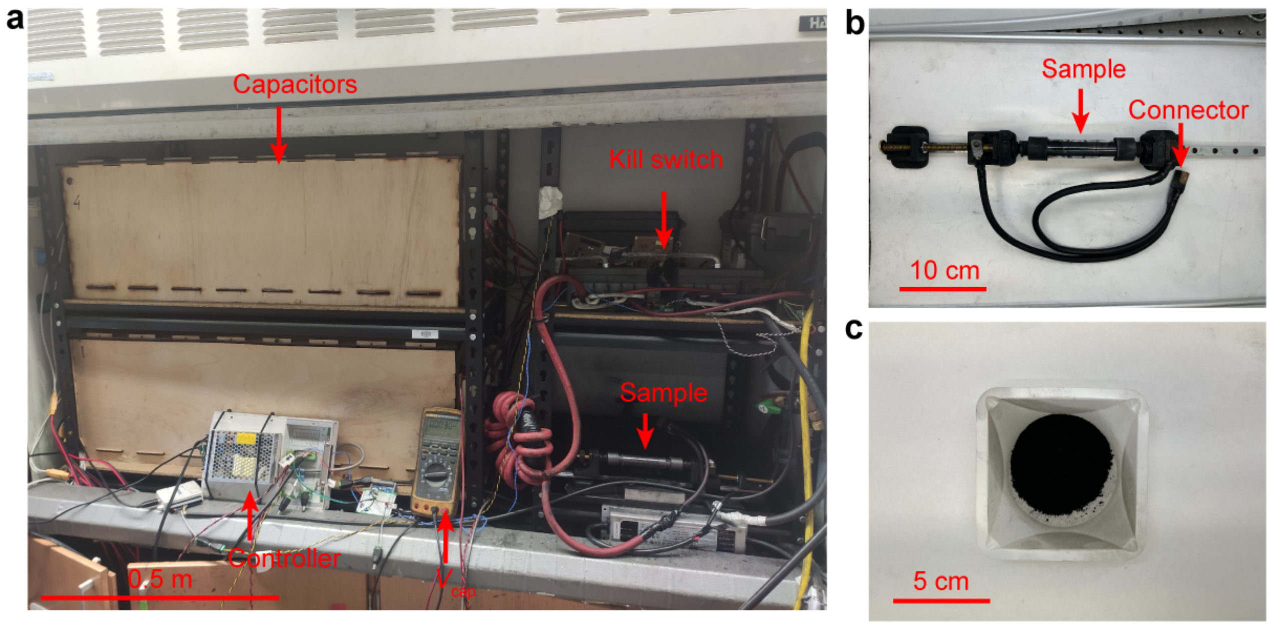

Supplementary Fig. 20. Scaling up of the FJH process. (**a**) Picture of the FJH setup with total capacitance of 0.624 F. (**b**) FJH reaction stage. (**c**) FJH sample size of 3 g per batch.

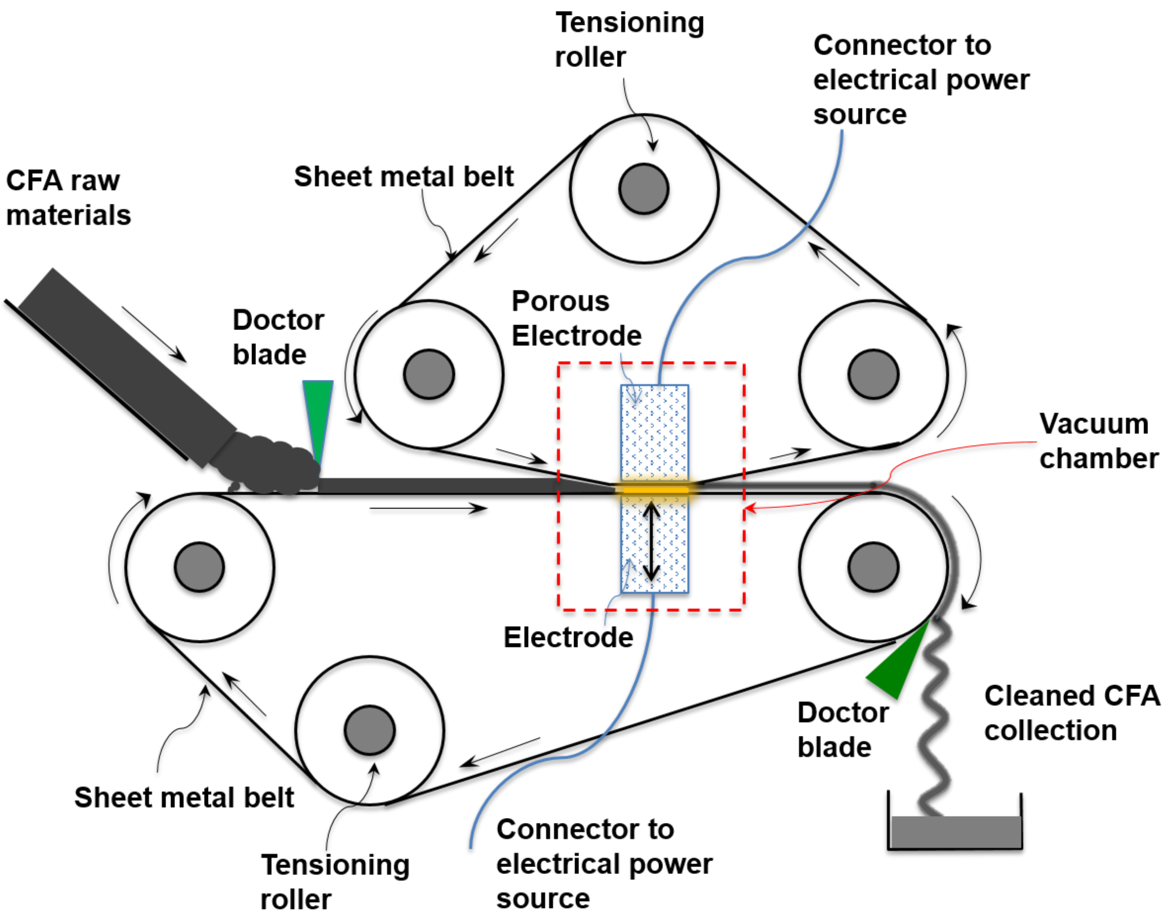

Supplementary Fig. 21. Conceptual design of a continuous FJH reactor. A sheet metal belt controlled by the tensioning roller is used for converting the CFA raw materials. The doctor blade is used to control the thickness and the compactness of the CFA raw materials. During FJH, since the sheet metal is not a good electrical conductor along its length, the current will be concentrated where the electrodes are located. The FJH zone is placed in a vacuum chamber to collect the volatiles.

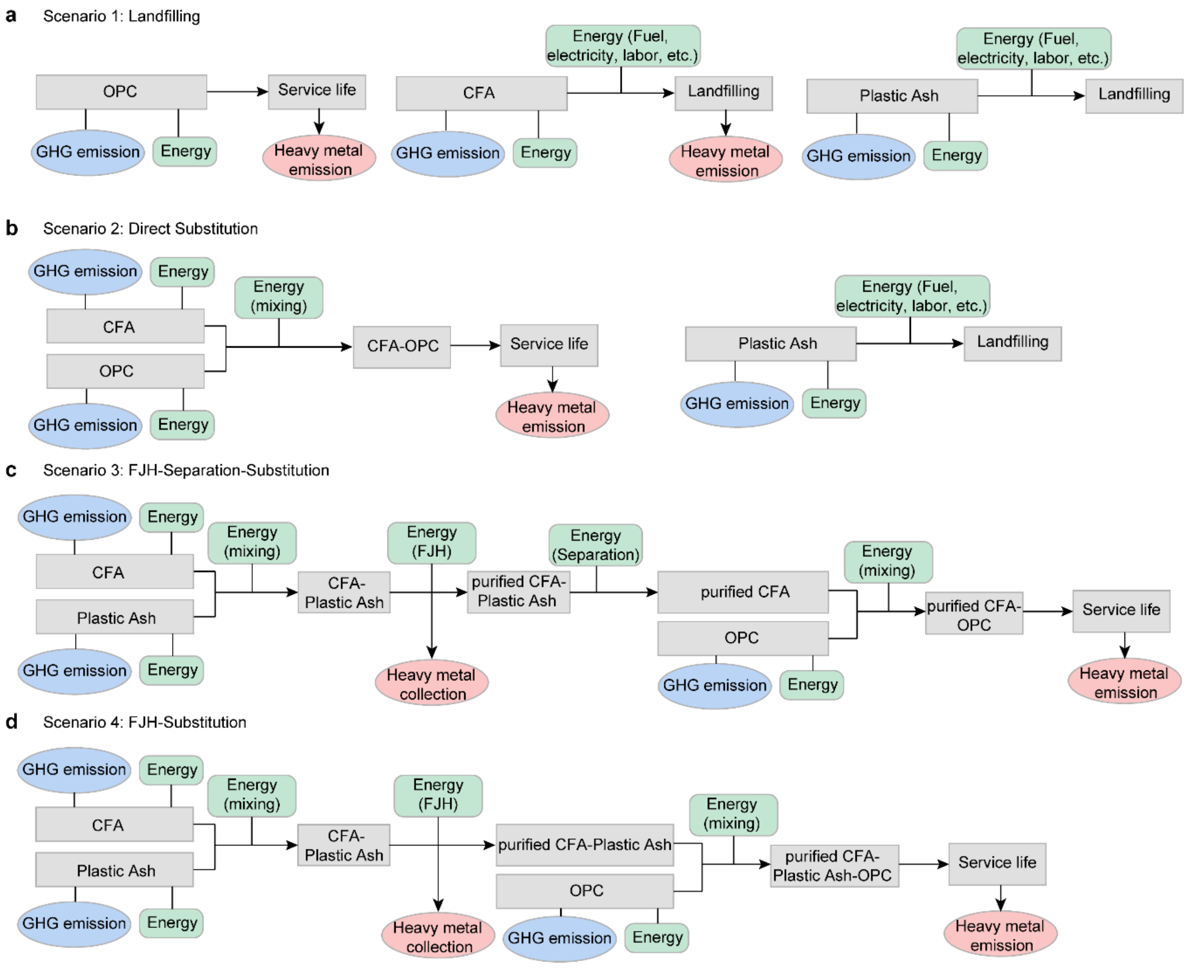

Supplementary Fig. 22. Flow chart representations and boundary conditions of the LCA models. (**a**) Scenario 1: Landfilling. OPC, ordinary Portland cement. CFA, coal fly ash. GHG, greenhouse gas. (**b**) Scenario 2: Direct Substitution. CFA, coal fly ash. (**c**) Scenario 3: FJH-Separation-Substitution. FJH, flash Joule heating. (**d**) Scenario 4: FJH-Substitution.

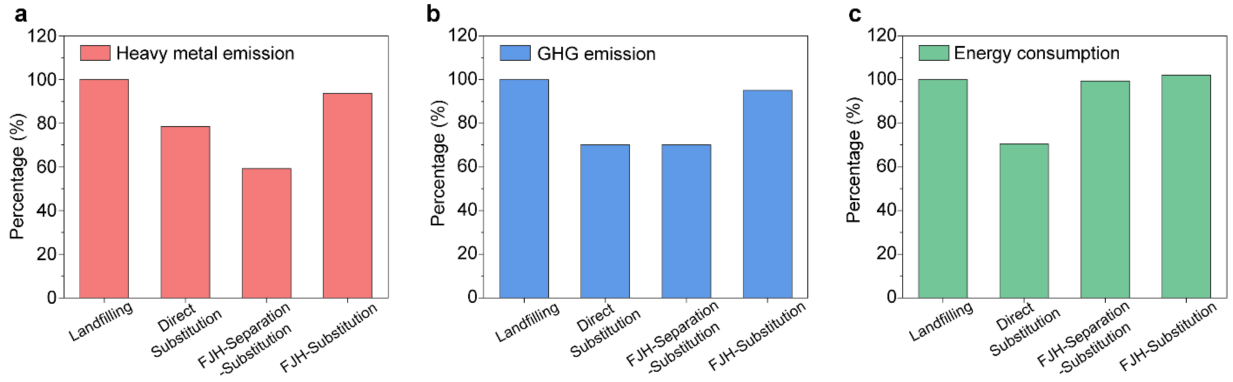

Supplementary Fig. 23. Environmental impact assessment. (**a**) Heavy metal emission percentage of various scenarios normalized to Landfilling. FJH, flash Joule heating. (**b**) Greenhouse gas (GHG) emission percentage of various scenarios normalized to Landfilling. (**c**) Energy consumption percentage of various scenarios normalized to Landfilling.

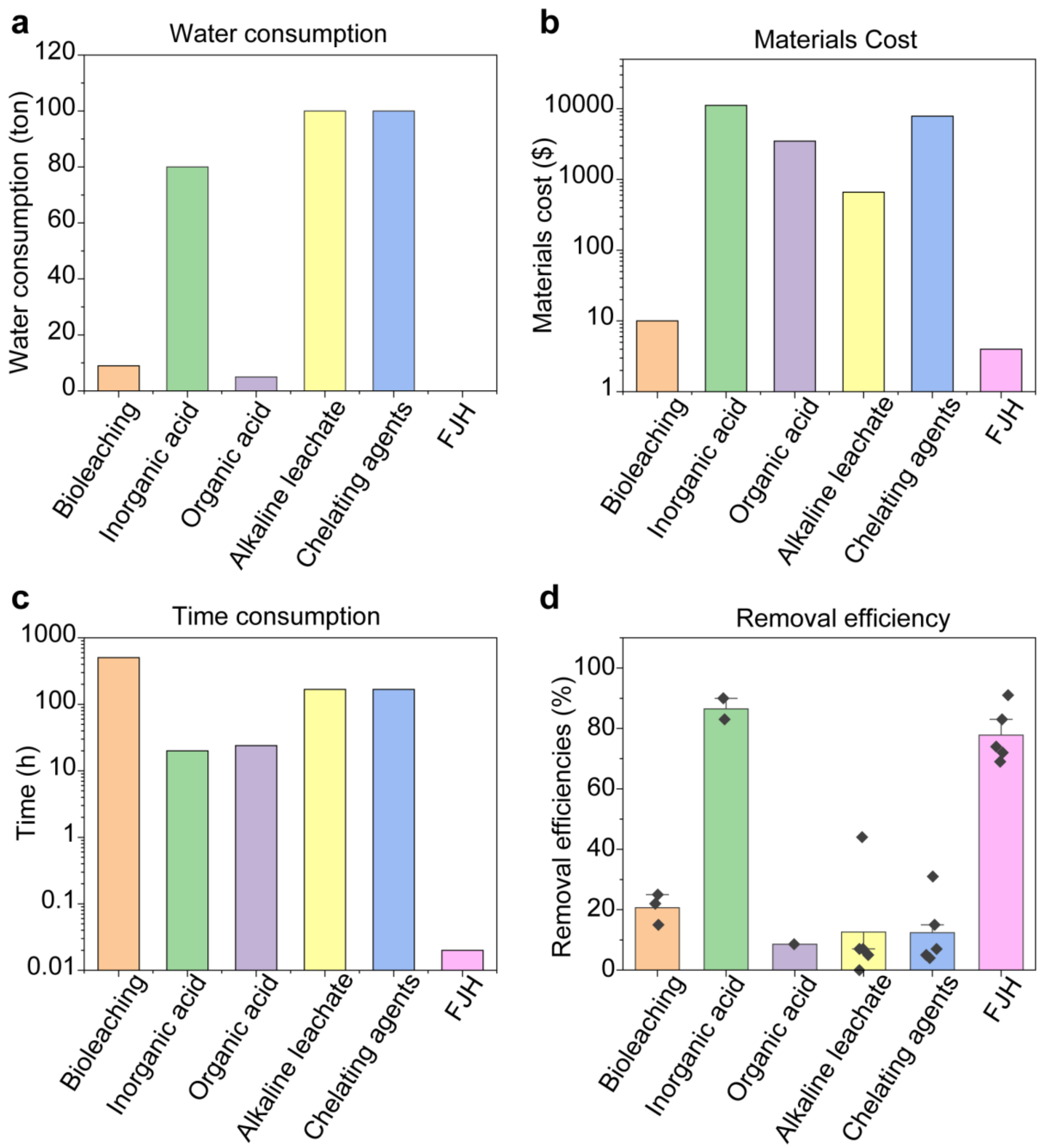

Supplementary Fig. 24. Comparison of the flash Joule heating (FJH) method with existing methods for heavy metal removal from CFA. (**a**) Water consumption per ton of CFA. (**b**) Materials cost per ton of CFA. (**c**) Time consumption. (**d**) Removal efficiencies.

Supplementary Table 1. Parameters for FJH.

Precursors	Mass Ratio	Mass (mg)	Resistance (Ω)	Voltage (V)	Time (s)	Mass after FJH (mg)
CFA-F:CB	2:1	150	1.0	60	1	125
CFA-F:CB	2:1	150	1.0	80	1	132
CFA-F:CB	2:1	150	1.0	100	1	58.4
CFA-F:CB	2:1	150	1.0	120	1	55.3
CFA-F:CB	2:1	150	1.0	150	1	43.7
CFA-C:CB	2:1	150	1.0	60	1	97.5
CFA-C:CB	2:1	150	1.0	80	1	90.0
CFA-C:CB	2:1	150	1.0	100	1	45.8
CFA-C:CB	2:1	150	1.0	120	1	50.2
CFA-C:CB	2:1	150	1.0	150	1	61.8
CFA-F:metcoke	2:1	150	1.5	120	1	81.2
CFA-F:Plastic Pyrolysis Ash	2:1	200	3.0	150	1	94.8
CFA-F-BC:CB	2:1	150	1.0	60	1	54.0
CFA-C-CWB:CB	2:1	150	1.0	60	1	80.0
BR:CB	2:1	150	1.0	60	1	97.5

Note: CFA-F: class F coal fly ash; CFA-C: class C coal fly ash; CB, carbon black; metcoke, metallurgical coke; BC, Boral Cumberland; CWB, Charah White Bluff; BR, bauxite residue; FJH, flash Joule heating.

Supplementary Table 2. Application of CFA in cement.

		OPC	OPC with 30 wt% raw CFA	OPC with 30 wt% purified CFA
Compressive strength (MPa)	28 day	41.7	60.8	62.8
	1 day	20.6	30.5	33.4
Modulus of elasticity (GPa)	28 day	20.0	23.8	25.5
	1 day	8.9	15.3	15.5

Note: CFA, coal fly ash; OPC, ordinary Portland cement.

Supplementary Table 3. Materials flow for various scenarios.

Scenarios	Landfilling (ton)	Direct Substitution (ton)	FJH-Separation-Substitution (ton)	FJH-Substitution (ton)
OPC (service life)	1	0.7	0.7	0.95
CFA (service life)	0	0.3	0.47	0.07
Plastic Ash (service life)	0	0	0.23	0.04
Purified CFA (service life)	0	0	0.3	0.05
Mixing (CFA+OPC)	0	1	1	1
Mixing (CFA+Plastic Ash)	0	0	0.7	0.11
FJH (CFA+Plastic Ash)	0	0	0.7	0.11
Separation (purified CFA+Plastic Ash)	0	0	0.333	0
CFA (landfill)	0.47	0.17	0	0.4
Plastic Ash (landfill)	0.23	0.23	0	0.19

Note: [a]The materials mass flow is normalized to 1 ton of cementitious materials in service life. [b]OPC, ordinary Portland cement; CFA, coal fly ash; FJH, flash Joule heating.

Supplementary Table 4. Life cycle inventory.

Impact Category	GHG emission (kg)	Heavy metal emission (g)	Energy consumption (MJ)
OPC (service life)	849.4969	2.24593	4581
CFA (service life)	10.1	1.8788	199
Plastic Ash (service life)	0.028	0	5.94
Purified CFA (service life)	0	0.93593	0
Mixing (CFA+OPC)	0	0	9.432
Mixing (CFA+Plastic Ash)	0	0	9.432
FJH (CFA+Plastic Ash)	0	0	2901.6
Separation (purified CFA+Plastic Ash)	0	0	4
CFA (landfill)	0	1.8788	19.6
Plastic Ash (landfill)	0	0	19.6

Note: [a]The environmental impacts or energy demands are normalized to production, processing, or landfilling of 1 ton of materials. [b]OPC, ordinary Portland cement; CFA, coal fly ash; FJH, flash Joule heating; GHG, greenhouse gas.

Supplementary Table 5. Heavy metal emissions for various scenarios.

Scenarios	Landfilling (g)	Direct Substitution (g)	FJH-Separation-Substitution (g)	FJH-Substitution (g)
OPC (service life)	2.24593	1.572151	1.572151	2.13363
CFA (service life)	0	0.56364	0	0
Plastic Ash (service life)	0	0	0	0
Purified CFA (service life)	0	0	0.28	0.0468
Mixing (CFA+OPC)	0	0	0	0
Mixing (CFA+Plastic Ash)	0	0	0	0
FJH (CFA+Plastic Ash)	0	0	0	0
Separation (purified CFA+Plastic Ash)	0	0	0	0
CFA (landfill)	0.883	0.319	0	0.75152
Plastic Ash (landfill)	0	0	0	0
SUM	3.13	2.45	1.85	2.93

Note: [a]The materials mass flow is normalized to 1 ton of cementitious materials in service life. [b]OPC, ordinary Portland cement; CFA, coal fly ash.

Supplementary Table 6. GHG emissions for various scenarios.

Scenarios	Landfilling (kg)	Direct Substitution (kg)	FJH-Separation-Substitution (kg)	FJH-Substitution (kg)
OPC (service life)	849.4969	594.647	594.647	807.022
CFA (service life)	0	3.03	4.747	0.707
Plastic Ash (service life)	0	0	0.00644	0.00112
Purified CFA (service life)	0	0	0	0
Mixing (CFA+OPC)	0	0	0	0
Mixing (CFA+Plastic Ash)	0	0	0	0
FJH (CFA+Plastic Ash)	0	0	0	0
Separation (purified CFA+Plastic Ash)	0	0	0	0
CFA (landfill)	4.747	1.717	0	4.04
Plastic Ash (landfill)	0.00644	0.00644	0	0.00532
SUM	854	599	599	812

Note: [a]The materials mass flow is normalized to 1 ton of cementitious materials in service life. [b]OPC, ordinary Portland cement; CFA, coal fly ash; FJH, flash Joule heating; GHG, greenhouse gas.

Supplementary Table 7. Energy consumption for various scenarios.

Scenarios	Landfilling (MJ)	Direct Substitution (MJ)	FJH-Separation-Substitution (MJ)	FJH-Substitution (MJ)
OPC (service life)	4581	3207	3207	4351.95
CFA (service life)	0	59.7	93.53	13.93
Plastic Ash (service life)	0	0	1.3662	0.2376
Purified CFA (service life)	0	0	0	0
Mixing (CFA+OPC)	0	0	9.432	9.432
Mixing (CFA+Plastic Ash)	0	0	6.6024	1.0375
FJH (CFA+Plastic Ash)	0	0	1340.64	319.176
Separation (purified CFA+Plastic Ash)	0	0	1.332	0
CFA (landfill)	102.742	37.162	0	87.44
Plastic Ash (landfill)	5.8742	5.8742	0	4.8526
SUM	4690	3310	4660	4788

Note: [a]The materials mass flow is normalized to 1 ton of cementitious materials in service life. [b]OPC, ordinary Portland cement; CFA, coal fly ash; FJH, flash Joule heating.

Supplementary Table 8. Comparison of methods for heavy metal removal from CFA.

Method	Reaction conditions	Materials Consumption per ton of CFA	Materials cost	Metals removed	Removal efficiency (%)
Bioleaching[12]	*T. thiooxidans* bacteria; FAD[a] (10% w/v); 3 weeks; 28 °C; 200 rpm	Water: ~9 tons<br>Chemicals: ~10 g of H_2SO_4<br>Others: *T. thiooxidans*	Water: ~$9.8<br>Chemicals: ~$0.06<br>Total: ~$10	Al; Fe	25; 15-22
Inorganic acid leaching[13]	0.1 M HNO_3; L/S[b] (80-100); 24 °C; 20 h	Water: 80-100 tons<br>Chemicals: 0.50-0.63 ton of HNO_3	Water: $86.8-108.5<br>Chemicals: $9750-12285<br>Total: ~$11120	As	83-90
Organic acid leaching[14]	citric acid, oxalic acid and gluconic acid (0.0317 + 0.0266 + 0.0625 M); L/S (5); 30 °C; 24 h; 220 rpm	Water: 5 tons<br>Chemicals: 30 kg of critic acid, 12 kg of oxalic acid, 61 kg of gluconic acid	Water: ~$5.4<br>Chemicals: ~$3472<br>Total: ~$3480	Cr	8.6
Alkaline leachate[15]	NH_3 (10 mM); L/S (100); 7 days; pH 7.4	Water: 100 tons<br>Chemicals: 17 kg of NH_3	Water: ~$108.5<br>Chemicals: ~$552<br>Total: ~$661	Mn; V; Cu; Ni; Cr;	0; 44; 7; 5; 7
Chelating agent[15]	Histidine (10 mM); L/S (100); 7 days; pH 7.4	Water: 100 tons<br>Chemicals: 155 kg of Histidine	Water: ~$108.5<br>Chemicals: ~$7750<br>Total: ~$7860	Mn; V; Cu; Ni; Cr	15; 31; 7; 4; 5
FJH, this work	Mixing with metcoke; FJH at 60-150 V; 1 s; Separation by sieving	Water: 0<br>Chemicals: 25 kg of metcoke	Water: 0<br>Chemicals: $3.8<br>Total: $3.8	As, Cd, Co, Ni, Pb	91; 83; 74; 69; 72

Note: [a]FAD: fly ash pulp density; [b]L/S: liquid/solid ratio; [c]Materials prices: industrial water ($3.10 per 100 cubic ft, or $1.085 per ton, ref[16]), H_2SO_4 ($5.8 per kg, ref[17]), HNO_3 ($19.5 per kg, ref[18]), citric acid ($14.3 per kg, ref[19]), oxalic acid ($85.8 per kg, ref[20]), gluconic acid ($33 per kg, ref[21]), ammonium hydroxide 28-30% solution ($30.9 per gallons, ref[22]), Histidine ($50 per kg, ref[23]), and MetCoke ($150 per ton, ref[24]); [d]CFA, coal fly ash; metcoke, metallurgical coke; FJH, flash Joule heating.

Supplementary References

1 Deng, B. *et al.* Urban mining by flash Joule heating. *Nat. Commun.* **12**, 5794 (2021).

2 Luong, D. X. *et al.* Gram-scale bottom-up flash graphene synthesis. *Nature* **577**, 647-651 (2020).

3 https://www.alibaba.com/showroom/metallurgical-coke-price.html, accessed July 10, 2022.

4 ISO, 2006. Life Cycle Assessment-Requirements and Guidelines (ISO 14044:2006).

5 https://greet.es.anl.gov/, accessed July 9th, 2022.

6 Teixeira, E. R., Mateus, R., Camões, A. F., Bragança, L. & Branco, F. G. Comparative environmental life-cycle analysis of concretes using biomass and coal fly ashes as partial cement replacement material. *J. Clean. Prod.* **112**, 2221-2230 (2016).

7 Nabavi-Pelesaraei, A., Bayat, R., Hosseinzadeh-Bandbafha, H., Afrasyabi, H. & Chau, K.-w. Modeling of energy consumption and environmental life cycle assessment for incineration and landfill systems of municipal solid waste management - A case study in Tehran Metropolis of Iran. *J. Clean. Prod.* **148**, 427-440 (2017).

8 https://www.lfatabletpresses.com/vh-powder-mixer, accessed July 9th, 2022.

9 Luong, D. X. *et al.* Gram-scale bottom-up flash graphene synthesis. *Nature* **577**, 647-651 (2020).

10 Stanford, M. G. *et al.* Flash Graphene Morphologies. *ACS Nano* **14**, 13691-13699 (2020).

11 Deng, B. *et al.* Rare earth elements from waste. *Sci, Adv.* **8**, eabm3132 (2022).

12 Seidel, A., Zimmels, Y. & Armon, R. Mechanism of bioleaching of coal fly ash by Thiobacillus thiooxidans. *Chem. Eng. J.* **83**, 123-130 (2001).

13 Xu, Y. H., Nakajima, T. & Ohki, A. Leaching of arsenic from coal fly ashes 1. leaching behavior of arsenic and mechanism study. *Toxicol. Environ. Chem.* **81**, 55-68 (2001).

14 Pangayao D, Gallardo S, editors (2014) Leaching of chromium from coal ash using citric acid, oxalic acid and gluconic acid by batch leaching procedure. In: IEEE 2014 International Conference on Humanoid, Nanotechnology, Information Technology, Communication and Control, Environment and Management (HNICEM).

15 Harris, W. R. & Silberman, D. Time-dependent leaching of coal fly ash by chelating agents. *Environ. Sci. Technol.* **17**, 139-145 (1983).

16 https://www.cityofbryan.net/commercialindustrial-metering-rates/, accessed Dec 27th, 2022.

17 https://www.laballey.com/products/sulfuric-acid-acs-grade?variant=40875821269147, accessed Dec. 27, 2022.

18 https://www.laballey.com/products/nitric-acid-solution-67-of-lab-grade?variant=7219028459579, accessed Dec. 27, 2022.

19 https://www.laballey.com/products/citirc-acid-anhydrous-food-grade?variant=7218973442107, accessed Dec. 27, 2022.

20 https://www.laballey.com/products/oxalic-acid-crystal-acs?variant=7219031277627, accessed Dec. 27, 2022.

21 https://www.alibaba.com/product-detail/Best-Price-CAS-527-07-1_1600560138233.html?s=p, accessed Dec. 27, 2022.

22 https://www.laballey.com/products/ammonium-hydroxide-acs?variant=40864633454747, accessed Dec. 27, 2022.

23 https://www.alibaba.com/product-detail/Dietary-supplement-bulk-l-histidine-l_1600435645833.html?spm=a2700.galleryofferlist.0.0.246d7a1fZSd4AU, accessed Dec. 27, 2022.

24 https://www.alibaba.com/product-detail/Semi-Coke-instead-of-Metallurgical-coke_1600088161385.html?spm=a2700.galleryofferlist.normal_offer.d_title.4b5e34cekU7eT4, accessed Dec. 27, 2022.
